# Isolation and characterization of novel acetogenic *Moorella* strains for employment as potential thermophilic biocatalysts

**DOI:** 10.1093/femsec/fiae109

**Published:** 2024-08-08

**Authors:** Tim Böer, Lisa Engelhardt, Alina Lüschen, Lena Eysell, Hiroki Yoshida, Dominik Schneider, Largus T Angenent, Mirko Basen, Rolf Daniel, Anja Poehlein

**Affiliations:** Genomic and Applied Microbiology and Göttingen Genomics Laboratory, Institute of Microbiology and Genetics, Georg-August-University Göttingen, 37077 Göttingen, Germany; Microbiology, Institute of Biological Sciences, University Rostock, 18059 Rostock, Germany; Genomic and Applied Microbiology and Göttingen Genomics Laboratory, Institute of Microbiology and Genetics, Georg-August-University Göttingen, 37077 Göttingen, Germany; Genomic and Applied Microbiology and Göttingen Genomics Laboratory, Institute of Microbiology and Genetics, Georg-August-University Göttingen, 37077 Göttingen, Germany; Environmental Biotechnology Group, Department of Geosciences, University of Tübingen, 72074 Tübingen, Germany; Genomic and Applied Microbiology and Göttingen Genomics Laboratory, Institute of Microbiology and Genetics, Georg-August-University Göttingen, 37077 Göttingen, Germany; Environmental Biotechnology Group, Department of Geosciences, University of Tübingen, 72074 Tübingen, Germany; Microbiology, Institute of Biological Sciences, University Rostock, 18059 Rostock, Germany; Genomic and Applied Microbiology and Göttingen Genomics Laboratory, Institute of Microbiology and Genetics, Georg-August-University Göttingen, 37077 Göttingen, Germany; Genomic and Applied Microbiology and Göttingen Genomics Laboratory, Institute of Microbiology and Genetics, Georg-August-University Göttingen, 37077 Göttingen, Germany

**Keywords:** acetogen, biotechnology, carboxydotrophic hydrogenogen, *Moorella thermoacetica*, thermophage, thermophile

## Abstract

Thermophilic acetogenic bacteria have attracted attention as promising candidates for biotechnological applications such as syngas fermentation, microbial electrosynthesis, and methanol conversion. Here, we aimed to isolate and characterize novel thermophilic acetogens from diverse environments. Enrichment of heterotrophic and autotrophic acetogens was monitored by 16S rRNA gene-based bacterial community analysis. Seven novel *Moorella* strains were isolated and characterized by genomic and physiological analyses. Two *Moorella humiferrea* isolates showed considerable differences during autotrophic growth. The *M. humiferrea* LNE isolate (DSM 117358) fermented carbon monoxide (CO) to acetate, while the *M. humiferrea* OCP isolate (DSM 117359) transformed CO to hydrogen and carbon dioxide (H_2_ + CO_2_), employing the water–gas shift reaction. Another carboxydotrophic hydrogenogenic *Moorella* strain was isolated from the covering soil of an active charcoal burning pile and proposed as the type strain (ACPs^T^) of the novel species *Moorella carbonis* (DSM 116161^T^ and CCOS 2103^T^). The remaining four novel strains were affiliated with *Moorella thermoacetica* and showed, together with the type strain DSM 2955^T^, the production of small amounts of ethanol from H_2_ + CO_2_ in addition to acetate. The physiological analyses of the novel *Moorella* strains revealed isolate-specific differences that considerably increase the knowledge base on thermophilic acetogens for future applications.

## Teaser

Isolation and characterization of novel gas-fermenting *Moorella* strains.

## Introduction

Due to their metabolic flexibility, acetogens have attracted attention as potential microbial production platforms for biotechnological applications. These range from the fermentation of industrial waste gases (syngas, which is a mixture of H_2_, CO_2_, and CO) (Bengelsdorf et al. 2018) to other abundant and cheap substrates such as methanol (Cotton et al. 2020) or lignocellulosic carbohydrates (Rahayu et al. 2017, Nguyen and Rabemanolontsoa 2022). Additionally, some acetogens were shown to utilize hydrogen (H_2_) that was produced at electrodes in a process called microbial electrosynthesis (Nevin et al. 2011). These technologies offer several advantages for the establishment of a sustainable and circular economy by using cheap and abundant resources or even waste products. Simultaneously, most applications recycle the greenhouse gas carbon dioxide (CO_2_) into industrially relevant biocommodities and thereby contribute to the global efforts of preventing climate warming. *Moorella thermoacetica* (formerly *Clostridium thermoaceticum*) was the second acetogenic bacterium isolated in 1942 by Fontaine et al. (1942) from horse manure. Wood and Ljungdahl used this microbe to elucidate the reactions of the reductive acetyl-CoA pathway, also called the Wood–Ljungdahl pathway (WLP) (Ragsdale 1997). The pathway consists of a methyl branch reducing CO_2_ to a methyl group and a carbonyl branch reducing CO_2_ to a carbonyl group. Both branches converge in the formation of acetyl-CoA, which is subsequently transformed to the fermentation product acetate. Reducing equivalents are provided for this reaction by an electron-bifurcating hydrogenase, oxidizing H_2_. The WLP is an energy-neutral pathway and energy conservation is achieved by building a chemiosmotic ion gradient via a membrane-bound complex consuming the reducing equivalents. The ion gradient is subsequently utilized by an ATP synthase for the conservation of energy in the form of adenosine triphosphate (ATP). The genus *Moorella* has been established in 1994 and hitherto described species comprise *M. thermoacetica* (Fontaine et al. 1942), *Moorella glycerini* (Slobodkin et al. 1997), *Moorella mulderi* (Balk et al. 2003), *Moorella humiferrea* (Nepomnyashchaya et al. 2012), *Moorella stamsii* (Alves et al. 2013), *Moorella sulfitireducens* (Slobodkina et al. 2022), and *Moorella caeni* (Vecchini Santaella et al. 2023). Autotrophic growth on H_2_ + CO_2_ has been described for *M. thermoacetica* (Kerby and Zeikus 1983) and *M. mulderi* (Balk et al. 2003). *Moorella sulfitireducens* was described to utilize H_2_ only as an electron donor when sulfite was provided as an electron acceptor (Slobodkina et al. 2022). Autotrophic growth on CO has been described for *M. thermoacetica* (Kerby and Zeikus 1983), *M. glycerini* (Alves et al. 2013), *M. stamsii* (Alves et al. 2013), *M. sulfitireducens* (Slobodkina et al. 2022), and *M. caeni* (Vecchini Santaella et al. 2023). Only *M. humiferrea* was described to not grow autotrophically (Nepomnyashchaya et al. 2012, Alves et al. 2013). The major fermentation product during autotrophic growth of acetogenic *Moorella* species was acetate; however, the strains *Moorella* sp. HUCC22-1 and *M. thermoacetica* Y72 were shown to also produce small amounts of ethanol when grown on H_2_ + CO_2_ (Sakai et al. 2004, Kimura et al. 2016). *Moorella thermoacetica* Y72 was additionally able to produce small amounts of glycerol. The species *M. stamsii* and *M. caeni* do not produce organic acids as fermentation products during autotrophic growth and instead employ the water–gas shift reaction, transforming CO to H_2_ and CO_2_. Characteristically, bacteria from the genus *Moorella* show optimal growth at temperatures around 60°C and therefore represent one of few examples for thermophilic acetogens. Other examples of thermophilic acetogens are *Thermoanaerobacter kivui, Thermacetogenium phaeum*, and the most recently isolated *Aceticella autotrophica* (Leigh et al. 1981, Hattori et al. 2000, Frolov et al. 2023). In contrast to mesophilic acetogens utilizing the Rnf complex for energy conservation, thermophilic acetogens have been shown to conserve energy during autotrophic growth by employing a membrane-bound energy-converting hydrogenase, the Ech complex (Schuchmann and Müller 2014, Katsyv and Müller 2022, Frolov et al. 2023). Two variants of the Ech complex were found in thermophilic acetogens. The Ech1 complex shows similarities to the bacterial formate hydrogenlyase complex, while the Ech2 complex shows similarities to archaeal hydrogenase systems (Hess et al. 2014). The elevated growth temperatures of thermophilic acetogens could provide several advantages in biotechnological processes, due to lowered costs for reactor cooling facilitated retrieval of volatile products, and lower contamination risks (Wiegel et al. 1985, Taylor et al. 2009, Bisaria and Kondo 2014). Furthermore, the genus *Moorella*, together with the exclusively acetogenic genus *Sporomusa*, is the only acetogenic bacterium described to possess both cytochromes and quinones (Rosenbaum and Müller 2021). This is of particular importance as cytochromes and quinones are hypothesized to enable additional routes for energy conservation as potential alternatives to the Ech complex (Rosenbaum and Müller 2023). Possible alternative energy-conserving systems utilizing cytochromes or quinones are the hetereodisulfide reductase subunits together with both subunits of the methylene-THF reductase (metFVHdrABCMvhD complex) and the fixABCX complex (Rosenbaum and Müller 2021, Kremp et al. 2022). Alternative energy conservation systems in the genus *Moorella* could provide a considerable advantage in terms of energy efficiency over cytochrome-free and quinone-free acetogens. Furthermore, most acetogenic *Moorella* species naturally possess the ability to grow under a 100% CO atmosphere, without the requirement for prior adaptation to CO as required for *T. kivui* (Weghoff and Müller 2016). Potential biotechnological applications of *Moorella* strains include the fermentation of (lignocellulosic) carbohydrates (Ehsanipour et al. 2016, Rahayu et al. 2017, Nguyen and Rabemanolontsoa 2022), the fermentation of synthesis gas (Sakai et al. 2005, Jia et al. 2023, Kato et al. 2024), and microbial electrosynthesis (Shin et al. 2003, Yu et al. 2017, Chen et al. 2018a, Ha et al. 2022). Additionally, acetogenic bacteria showed the highest energetic efficiency for the conversion of methanol or formate. Hence, they are optimal candidates for biocatalysts employed in an economy based on these one-carbon substrates as feedstocks (Claassens et al. 2019, Müller 2019, Cotton et al. 2020). Here, we isolated novel *Moorella* strains from diverse environmental samples and evaluated their applicability for biotechnological applications by physiological and genomic characterizations.

## Methods

### Enrichment, isolation, and cultivation of *Moorella* strains

Enrichment cultures were prepared with a variety of different media compositions, substrates, and inocula under sterile and anaerobic conditions (Table 1). For enrichments performed at the University of Göttingen (isolates KAM, BGP, COM, LNE, OCP, and ACPs), the following cultivation scheme was conducted. Environmental samples were dissolved (50% w/v) in phosphate buffered saline (PBS) buffer (NaCl, 8 g/l; KCl, 0.2 g/l; Na_2_HPO_4_, 1.42 g/l; KH_2_PO_4_, 0.24 g/l) and selected inocula were additionally pasteurized at 80°C for 10 min to select for spore-forming bacteria. Enrichment cultures were inoculated with 20 µl of this solution. Cultivations were conducted using the media recipes provided by the German Collection of Microorganisms and Cell Cultures (DSMZ, Braunschweig, Germany) DSM 60 (D-medium) and DSM 879 (C-medium), as well as the media described in Redl et al. (2020) (M-medium) and Groher and Weuster-Botz (2016) (G-medium). Carbon sources and complex substrates were omitted from the media, and yeast extract was reduced to concentrations ranging from 0.05 to 2 g/l to reduce the growth of amino acid-fermenting bacteria. Autotrophic enrichments were performed in 500 ml Afnor flasks (Ochs, Bovenden, Germany) sealed with a butyl rubber septum and filled with 100 ml medium sparged for 2 min with a H_2_ + CO_2_ (66%/33%) mixture adding a final overpressure of 2 bar. Heterotrophic enrichments were performed in Hungate tubes with 10 ml medium and vanillate (5 mM), syringate (5 mM), *N,N*-dimethylglycine (DMG) (30 mM), or betaine (30 mM) as carbon sources. Vanillate and syringate were added to the media prior to autoclaving, while DMG and betaine were added from sterile anaerobic stock solutions after autoclaving the media. All enrichments were performed at 60°C–65°C and autotrophic cultures were incubated horizontally to increase the interface between medium and atmosphere. The isolate MBA was enriched at the University of Rostock with the following cultivation approach. The compost sample was resuspended (0.1%, w/v) in the modified DSM 171 medium, which was described in Basen et al. (2018) (T-medium) and supplemented with 2-bromoethanesulfonate (BES). The resuspension was transferred in a volume of 20 ml to 500 ml Afnor flasks filled with 180 ml T-medium and methanol (100 mM) as a substrate. The culture was incubated at 60°C at 150 rpm and transferred four times every 48 h as described for the transfer of the resuspension. All enriched *Moorella* strains were isolated by cultivation in M-medium on methanol or CO (50%, v/v), diluted in N_2_/CO_2_ (80%/20%) as a substrate and subsequent plating on M-medium plates with GELRITE (10 g/l) (Roth, Karlsruhe, Germany) and fructose (30 mM). Colonies were singularized after 1 week of incubation in an anaerobic jar at 60°C and purified by dilution streaking twice. For isolates not growing on solid medium, serial dilutions were performed twice with cultures grown on CO using the highest dilution showing growth to inoculate the subsequent dilution series. After isolation, cultures were routinely grown in M-medium containing yeast extract (0.5 g/l) and fructose (30 mM) as a substrate inoculated to an OD_600_ of 0.01. The *Moorella* strains *M. thermoacetica* DSM 2955^T^, DSM 521^T^, and DSM 103132, and *M. humiferrea* DSM 23265^T^ were cultivated from lyophilisate obtained from the DSMZ. For the physiological characterizations, the temperature optima were investigated by incubation at temperatures ranging from 30°C to 75°C in 5°C intervals. NaCl and pH optima were investigated at the respective temperature optima of the strains. NaCl optima were determined by cultivation at NaCl concentrations ranging from 0% to 1% in 0.2% intervals and from 1% to 5% in 1% intervals. pH optima were obtained by setting the pH to 4–10 in 0.5 intervals, using HCl and NaOH. Gram staining was carried out following the Claus protocol (Claus 1992). Substrate utilization was evaluated by cultivation in Hungate tubes adding substrates from sterile anaerobic stock solutions to a final concentration of 30 mM. The utilization of electron acceptors was evaluated with glycerol (30 mM) and fructose (30 mM) as electron donors and sodium thiosulfate (30 mM), sodium sulfite (30 mM), sodium sulfate (30 mM), sodium nitrite (30 mM), sodium nitrate (30 mM), sodium perchlorate (30 mM), dimethyl sulfoxide (DMSO) (30 mM), fumarate (10 mM), and 9,10-anthraquinone-2,6-disulfonate (AQDS) (10 mM) as electron acceptors. Growth was measured throughout the course of 72 h with cultures without electron acceptors as negative control. For the physiological characterizations and substrate tests, OD measurements were performed in Hungate tubes at 600 nm and cultures in triplicates. Autotrophic growth tests in pure culture were performed in 500 ml Afnor flasks with 50 ml M-medium (yeast extract 0.5 g/l) modified, according to the optimal NaCl concentration and pH assessed for the respective strain. Cultures were incubated horizontally at the respective temperature optimum of the isolate and growth was measured at 600 nm in triplicates using the spectrophotometer WPA S800 (Biochrom, Berlin, Germany). *Moorella thermoacetica* DSM 2955^T^ was the best growing strain on H_2_ + CO_2_ and methanol in the study of Redl et al. (2020), and it was employed as a reference strain for growth cultivated in M-medium at 60°C. Cellular fatty acid profile analysis was performed with the identification service provided by DSMZ. Acetate and ethanol concentrations were determined by gas chromatography (GC), as described in Baum et al. (2024). Headspace concentrations of CO, CO_2_, and H_2_ during autotrophic growth were determined by GC using an SRI 8610C gas chromatograph (SRI Instruments, Earl St., Torrance, USA). The chromatograph was equipped with a thermal conductivity detector, a silica gel packed column (length 2 m, outer diameter 1/8 in.) (SRI Instruments), and a molecular sieve 13X packed column (length 2 m, outer diameter 1/8 in.) (SRI Instruments).

**Table 1.** Enrichment and isolation strategies conducted for the novel *Moorella* isolates.

Isolate	Inoculum	Medium	Substrate/temperature	Subcultivation	Isolation
KAM	Hot spring sediment, Kamchatka Peninsula	D-medium: omitting tryptone and glucose, reducing yeast extract to 2 g/l	H_2_ + CO_2_ (66%/33%)/65°C	Methanol (30 mM)	M-medium plates
BGP	Pasteurized sludge from biogas plant, Rosdorf, Germany	G-medium: reducing yeast extract to 0.05 g/l	H_2_ + CO_2_ (66%/33%)/60°C	CO (50%)	M-medium plates
COM	Pasteurized compost sludge from composting plant, Göttingen, Germany	C-medium: reducing yeast extract to 0.5 g/l	DMG (30 mM)/60°C	CO (50%)	M-medium plates
MBA	Compost sludge from waste management plant, Rostock, Germany	T-medium: adding 0.05 g/l yeast extract and BES (10 mM)	Methanol (100 mM)/60°C	CO (50%)	M-medium plates
LNE	Pasteurized Leine river sediment, Göttingen, Germany	M-medium	H_2_ + CO_2_ (66%/33%)/60°C	CO (50%)	CO dilution series
OCP	Pasteurized soil from beneath an old charcoal burning pile location, Hasselfelde, Germany	M-medium	Vanillate (5 mM)/60°C	CO (50%)	M-medium plates
ACPs	Pasteurized soil covering an active charcoal burning pile, Hasselfelde, Germany	M-medium	Vanillate (5 mM)/60°C	CO (50%)	M-medium plates

### Bacterial community analysis of enrichment cultures

Autotrophic enrichment cultures were sampled in a volume of 6 ml and heterotrophic enrichment cultures were sampled in a volume of 2 ml. Cell pellets were obtained by centrifugation at 18 000 *× g* for 2 min and subsequently dissolved in 150 µl PBS buffer. Initial cell lysis was performed by the addition of 5 µl of lysozyme (100 mg/ml) and incubation at 37°C for 30 min. Protein precipitation and nucleic acid purification were performed with the MasterPure Complete DNA and RNA Purification Kit (Epicentre, Madison, USA) following the manufacturer’s instructions for cell samples. The bacterial community was assessed by amplification and sequencing of the V3/V4 region of the 16S rRNA gene following the protocol as described in Berkelmann et al. (2020). Amplicon data analysis was performed with the NGS-4-ECOPROD pipeline (v0.1, https://github.com/dschnei1/ngs4ecoprod) (Schneider 2023). Briefly, quality filtering of raw sequencing data was performed with fastp (v0.23.2) (Chen et al. 2018b). Forward and reverse reads were trimmed using Cutadapt (v3.3 with PYTHON 3.7.3) (Martin 2011) and merged with VSEARCH (v2.12.0) (Rognes et al. 2016). All samples were combined into a single file using an inhouse bash script. VSEARCH (v2.12.0) (Rognes et al. 2016) and the pipeline UNOISE3 (Edgar and Flyvbjerg 2010) were utilized to perform size sorting and filtering of all sequences smaller than 300 bp, as well as denoising and dereplication of amplicon sequences. Chimera removal was performed using UCHIME3 (Edgar 2016), first with the *de novo* method (uchime3_denovo) and subsequently with the reference-based method (uchime_ref) using the SILVA SSU database (v138.1). Amplicon sequence variants were combined with read counts to create an abundance table and taxonomic assignments were obtained by using the Bayesian LCA-based taxonomical classification method (BLCA) with the SILVA SSU database (v138.1) (Gao et al. 2017). If the confidence score of an assigned taxonomy level fell below the 80% threshold, the respective taxonomic level was changed to unclassified. Relative abundances were visualized in RStudio using the packages ampvis2 (v2.7.31) (Andersen et al. 2018) and ggplot2 (v3.4.1) (Wickham 2016). Sequences from chloroplasts, eukaryote, mitochondria, and archaea were filtered out. Bacterial amplicon sequence variants (ASVs) with abundances under 2% were summarized under the term low abundance.

### Genome sequencing, assembly, and annotation

DNA was isolated as described for the bacterial community analysis. Illumina sequencing libraries were prepared using the Nextera XT DNA Sample Preparation Kit (Illumina, San Diego, CA, USA) and sequenced with a MiSeq system and v3 chemistry (600 cycles), following the instructions of the manufacturer (Illumina). The Nanopore sequencing libraries were prepared with 1.5 µg high molecular weight DNA, using the Ligation Sequencing Kit 1D (SQK-LSK109) and the native barcode expansion kit (EXP-NBD114), as recommended by the manufacturer (Oxford Nanopore Technologies, Oxford, UK). Nanopore sequencing was conducted for 72 h with the MinION device Mk1B, the SpotON flow cell R9.4.1, and the MinKNOW software (v22.10.7) following the instructions of the manufacturer (Oxford Nanopore Technologies). Demultiplexing and base calling of Nanopore sequencing data were performed with the Guppy software in high accuracy (HAC) mode (v6.4.2). Long-read *de novo* genome assemblies were performed following the suggested Trycycler workflow for bacterial genome assemblies by Wick et al. (2023). Briefly, quality control and adapter trimming of paired-end Illumina sequences were performed with fastp (v0.23.2) (Chen et al. 2018b) and Trimmomatic (v0.39; LEADING: 3, TRAILING: 3, SLIDINGWINDOW: 4:15, and MINLEN:50) (Bolger et al. 2014). Porechop (v0.2.4) was used for adapter trimming of Nanopore reads, which were subsequently filtered with Filtlong (v0.2.1) to a minimal read length of 1 kb, discarding the worst 5% of sequences in terms of quality. Long reads were subsampled 24 times with Trycycler (v0.5.3) (Wick et al. 2021) and eight subsets from each were used as input for the long-read assemblers Flye (v2.9.1) (Kolmogorov et al. 2019), Canu (v2.2) (Koren et al. 2017), and Raven (v1.8.1) (Vaser and Šikić 2021). Assemblies were combined to a single consensus sequence with Trycycler and the consensus sequence was polished with the full long-read data using Medaka (v1.5.0) and finally polished with the processed short-read sequences using Polypolish (v0.5.0) (Wick and Holt 2022). Genomes were reorientated to start with the *dnaA* gene using the fixstart function of Circlator (v1.5.5) (Hunt et al. 2015). Genome annotations were performed with Prokka (v1.14.6) (Seemann 2014) and quality assessment of the final genome assemblies was conducted with CheckM2 (v1.0.2) (Chklovski et al. 2023). Visualization of gene clusters were performed with clinker (v.0.0.28) (Gilchrist and Chooi 2021).

### Genome-based metabolic and phylogenomic analysis

The phylogenomic analysis was performed with *Moorella* genome sequences available at NCBI Genbank/JGI GOLD using pyani (v0.2.12) (Pritchard et al. 2016) and the MUMmer alignment option (Marçais et al. 2018) assessing average nucleotide identities (ANIm). The assembly accession numbers of analyzed reference genomes are listed in Table S1. Unique orthologous groups (OGs) were identified by reannotating every *Moorella* genome sequence with Prokka (v1.14.6) and subsequent orthology analysis using Proteinortho (v6.0.31) (Lechner et al. 2011) with an identity threshold of 50% and an e-value of 1e^−10^. Unique OGs were visualized in R with the ggplot2 package (v3.4.1) (Wickham 2016).

### Prophage activity screening

Prophage activity screenings for spontaneously released prophages were performed for all seven novel *Moorella* isolates. Furthermore, the following reference strains were investigated: *M. thermoacetica* DSM 521^T^, *M. thermoacetica* DSM 2955^T^, *M. thermoacetica* DSM 103132, and *M. humiferrea* DSM 23265^T^. Cultures were grown to the late exponential phase and harvested in a volume of 10 ml by centrifugation at 2600 × *g* for 5 min. The supernatant was sterile filtered with a 0.45-µm filter (Sarstedt, Nümbrecht, Germany) to remove remaining bacterial cells. Subsequently, 20 µl of lysozyme (100 mg/ml in ddH_2_O) (Serva, Heidelberg, Germany), 20 µl of DNase I (1 U/µl) (Epicentre), and 20 µl of RNase A (5 µg/µl) (Epicentre) were added to the solution and incubated at 37°C overnight. The following day, the solution was centrifuged at 36 000 × *g* for 2 h at room temperature in a DU PONT OTD 50B ultracentrifuge (Thermo Fisher Scientific, Waltham, USA). The supernatant was discarded and the resulting phage pellet was resuspended in 100 µl of TKM buffer [10 mM Tris–HCl (pH 7.5), 10 mM KCl, and 1 mM MgCl_2_] and stored at 4°C. For the DNA isolation from the dissolved phage pellets, the DNase I was first inactivated by incubation at 75°C for 10 min. Protein precipitation and nucleic acid purification from the solution were continued as described for the MasterPure Complete DNA and RNA Purification Kit (Epicentre) by the manufacturer’s instructions for cell samples. DNA was subjected to Illumina MiSeq sequencing and adapter removal was performed as described for genome sequencing. The obtained data were mapped to the respective reference genomes with Bowtie2 (v2.4.4) (Langmead and Salzberg 2012) and visualized with the BRIG software (v0.95) (Alikhan et al. 2011). SAM files of the Bowtie2 mapping were converted to coverage graphs with the respective BRIG module, using window size 1 and setting the maximal coverage graph value to 700. Prophage and genomic island predictions were added from PHASTEST (Wishart et al. 2023) and Island Viewer 4 (Bertelli et al. 2017). The nucleotide sequence of regions showing activity in the prophage screening was extracted and annotated with Pharokka (v1.3.2) (Bouras et al. 2023). Phage sequences were reorientated to begin with the large subunit terminase gene and plotted using the corresponding Pharokka functions.

## Results and discussion

### Bacterial community analysis of enrichment cultures

**Figure 1.**
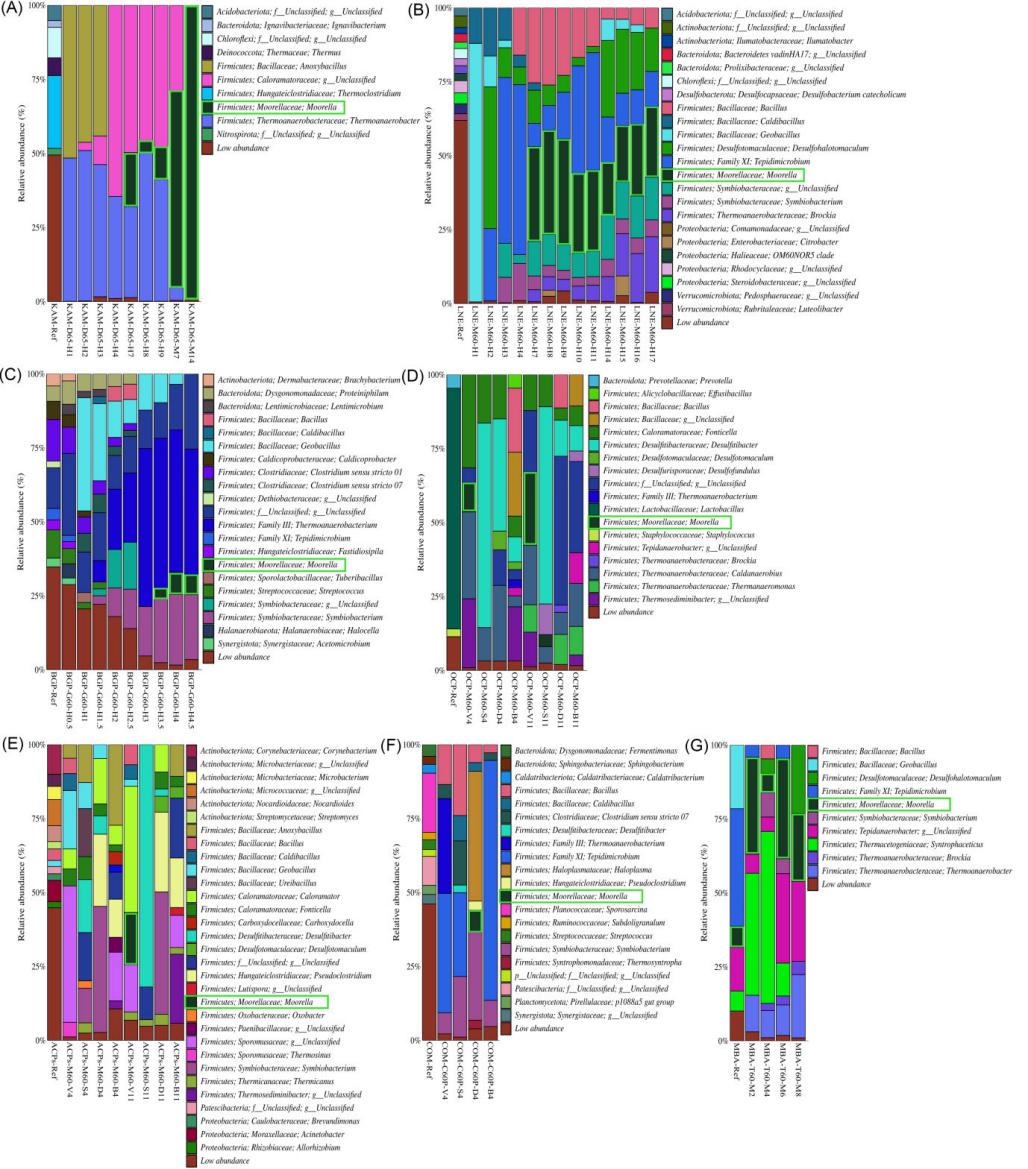
Bacterial community composition of seven thermophilic enrichment cultures by 16S rRNA gene-based amplicon analysis. The first samples for each enrichment panel (A–G) depict the assessed bacterial community of the inocula of the enrichment cultures (-Ref). The following sample code was utilized for the enrichment cultures: inoculum-medium temperature-substrate day. Media utilized comprise D-medium (D), M-medium (M), G-medium (G), C-medium (C), and T-medium (T). Incubation temperatures of 60°C and 65°C were utilized. The enrichments were performed with the substrates H_2_ + CO_2_ (H), methanol (M), vanillate (V), syringate (S), DMG (D), and betaine (B). The figure visualizes the enrichments from hot spring sediment from Kamchatka (Russia) (KAM, A), Leine river sediment from Göttingen (Germany) (LNE, B), sludge from the biogas plant in Rosdorf (Germany) (BGP, C), soil beneath an old charcoal pile location in Hasselfelde (Germany) (OCP, D), soil covering an active charcoal burning pile in Hasselfelde (Germany) (ACPs, E), compost sludge from the composting plant in Göttingen (Germany) (COM, F), and compost sludge from the waste management plant in Rostock (Germany) (MBA, G). The colour legend to the right of barcharts shows the assigned bacterial taxonomy on the phylum, family, and genus levels by BLCA. For phylogenetic levels with BLCA confidence scores lower than 80%, the bacterial phylum (p), family (f), or genus (g) was changed to unclassified. Bars from the genus *Moorella* are highlighted in green.

The environmental samples showed a very diverse and inoculum-specific bacterial community composition (left bar in Fig. 1A–G). 16S rRNA gene sequences corresponding to members of the genus *Moorella* were not detected in six of the seven environmental samples, with the exception of the resuspended compost sludge from Rostock (MBA_Ref; 7.12% *Moorella*). Nevertheless, *Moorella* was successfully enriched from every environmental sample. This implies that *Moorella* bacteria were present in low abundances under the detection threshold of the performed 16S rRNA amplicon surveying effort and potentially also present in the form of endospores. For the autotrophic enrichment cultures grown on H_2_ + CO_2_, bacteria from the genus *Moorella* were detected after 3.5–7 days of incubation (Fig. 1A–C). Heterotrophic enrichments with vanillate lead to *Moorella* growth after 4 days for the OCP enrichment and after 11 days for the ACPs enrichment (Fig. 1D and E). The highest relative *Moorella* abundance was detected after 11 days in both enrichments with 17.64% for ACPs and 24.64% for OCPs. Enrichments with syringate as a substrate only showed *Moorella* growth in the OCP enrichment after 11 days of incubation with a relative abundance of 4.04%. No growth of *Moorella* was detected in enrichments on betaine. DMG leads to a growth of 7.29% *Moorella* in the COM enrichment after 4 days (Fig. 1F). The enrichment culture on methanol showed a relative abundance of 32.80% for *Moorella* already in the first sample taken after 48 h (Fig. 1G). Besides the growth of *Moorella*, several nonacetogenic genera, which showed substrate-unspecific growth on the different media used for enrichments, were detected. Several genera from the family *Bacillaceae* were present, particularly in samples taken during the first days of enrichment. These bacteria utilize trace amounts of nitrite or nitrate derived from the oxidation of organic matter introduced by the inocula or the added yeast extract as terminal electron acceptors during anaerobic growth. (Nakano and Zuber 1998). Other abundant bacteria in the enrichments were members of the genera *Thermoanaerobacter, Thermoanaerobacterium, Tepidimicrobium, Clostridium sensu stricto* 7, *Desulfotomaculum, Desulfohalotomaculum, Fonticella, Caldanaerobius, Thermosediminibacter*, and *Pseudoclostridium*, and the families *Sporomusaceae, Caloramatoraceae*, and *Symbiobacteraceae*. These bacteria grew heterotrophically with the yeast extract employing the Stickland reaction (Slobodkin et al. 2006, Lee et al. 2008, Fraj et al. 2013). Furthermore, enrichments contained putative acetate-oxidizing syntrophs from the genera *Syntrophaceticus* and *Tepidanaerobacter* (Müller et al. 2013). Heterotrophic enrichment cultures also contained substrate-specific growth of likely nonacetogenic bacteria. For example, enrichments with syringate lead to high relative abundances of an uncultured bacterium from the genus *Desulfitibacter*. The most similar hit by BLASTn from NCBI was the uncultured bacterium MW-B37 that was isolated from water-flooded petroleum reservoirs from the Huabei Oilfield in China with 97.18% and the *Zhaonella formicivorans* type strain DSM 107278^T^ isolated from the Shengli Oilfield in China with 96.96% (Tang et al. 2012, Lv et al. 2020). The genus *Zhaonella* forms a monophyletic group with the genera *Calderihabitans, Desulfitibacter*, and *Moorella. Zhaonella formicivorans* was described to grow with betaine, formate, and methanol. Indeed, the uncultured bacterium enriched with syringate was also detected in enrichments with betaine and DMG. However, growth with syringate was not tested for *Z. formicivorans*. Although the draft genome contained a full set of genes for acetogenesis, *Z. formicivorans* was described as nonacetogenic as no growth occurred with H_2_ + CO_2_ as a substrate (Lv et al. 2020). Growth on methoxylated compounds has been mostly described for acetogens, while other bacteria usually require the presence of organic or inorganic electron acceptors such as fumarate, nitrate, or sulfate (Khomyakova and Slobodkin 2023). For heterotrophic cultivation of *Moorella*, methanol and vanillate were the most successful substrates in our enrichments. As syringate does contain one methoxyl group more than vanillate, it theoretically provides twice the energy for growth by *O*-demethylation. Pure cultures grown with syringate were shown to achieve higher optical densities than pure cultures grown with vanillate (Daniel et al. 1988). However, in our enrichment cultures, the employment of vanillate resulted in faster *Moorella* growth compared to syringate as substrate. This is likely caused by the facilitated accessibility of methyltransferases to the methoxyl group in vanillate than in syringate.

### Genomic characterization

#### Phylogenomic analysis

**Figure 2.**
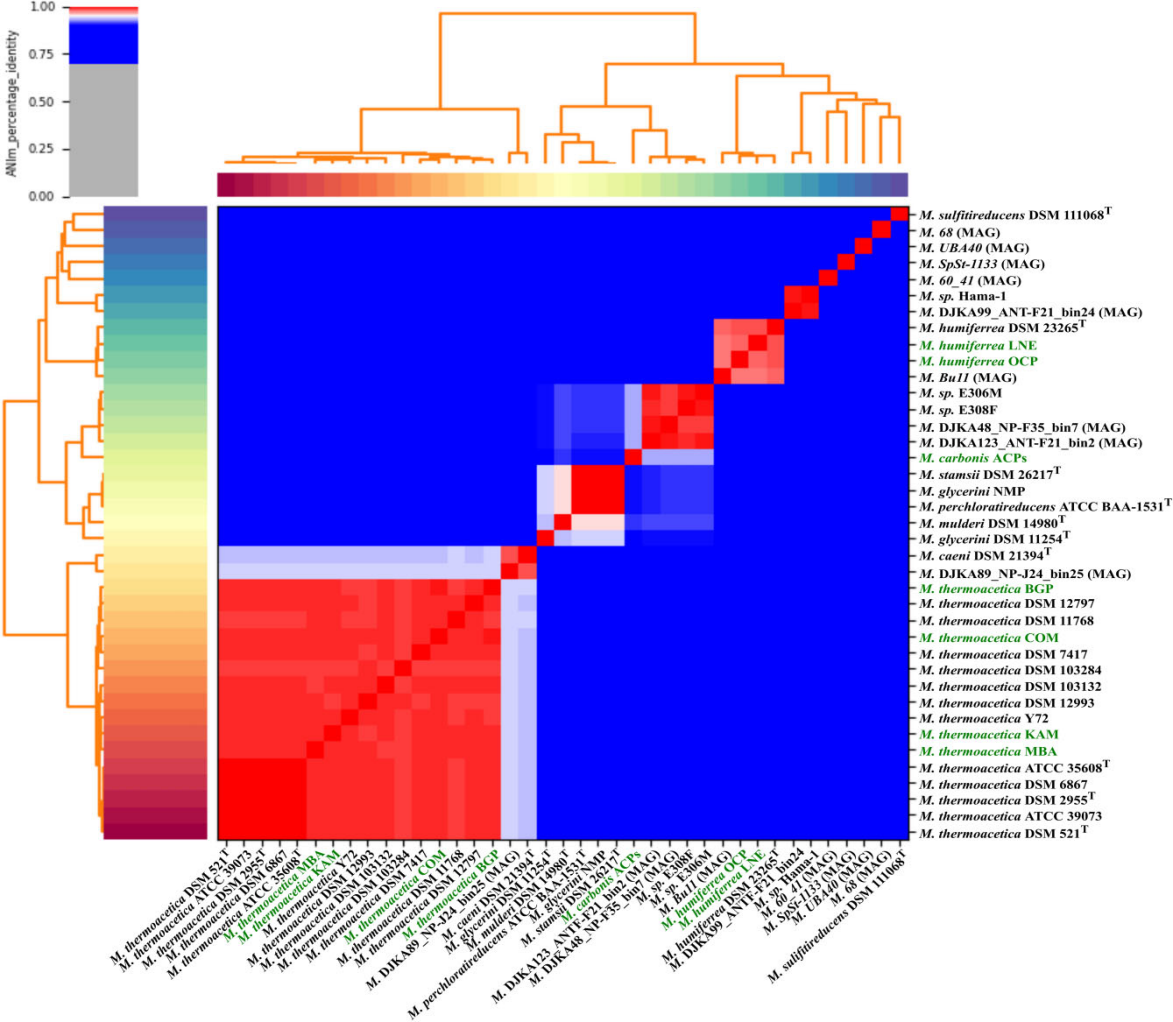
Phylogenomic analysis of novel and available *Moorella* genome sequences. The plot visualizes the ANIm percentage identity from 0% to 70% in grey, 70% to 95% in blue, and 95% to 100% in red. Genome sequences derived from metagenomic assembled genomes are marked by (MAG) and genome sequences of novel *Moorella* isolates from this study are highlighted in green. Assembly accession numbers of all genomes analyzed are listed in Table S1.

Genome features for the novel *Moorella* strains are summarized in Table S2. Genome assemblies of the novel *Moorella* isolates yielded single replicons with sizes ranging from 2.4 to 2.8 Mb. The GC content varied from 53% to 56%. CheckM2 assessed genome completeness of 99.75%–99.99% and contamination scores from 0% to 0.21%. All isolates encoded two complete rRNA gene clusters and at least one CRISPR repeat. To obtain a phylogenetic classification of the novel *Moorella* isolates, we performed a phylogenomic analysis with ANIm (Fig. 2). Genome sequences were compared between our *Moorella* isolates and *Moorella* genome sequences, including metagenomic assembled genomes (MAGs). As a threshold for species assignment, a percentage identity of 95% was applied (Goris et al. 2007). The majority of genome sequences formed a phylogenomic cluster corresponding to the species *M. thermoacetica*, including the novel *Moorella* isolates KAM, BGP, COM, and MBA. The *M. thermoacetica* strains DSM 521^T^, DSM 2955^T^, ATCC 35608^T^, DSM 6867, and ATCC 39079 were with more than 99.96% identity to each other more identical than other *M. thermoacetica* isolates. Other *M. thermoacetica* isolates showed highest identities ranging from 98.49% to 99.18%. Our isolates KAM, BGP, COM, and MBA were most identical to *M. thermoacetica* DSM 7417 with ANIm values of 99.04%, 98.97%, 99.06%, and 99.05%, respectively. Together with the Bu11 MAG, the isolates OCP and LNE formed a phylogenomic cluster corresponding to the species *M. humiferrea. Moorella humiferrea* LNE showed an identity of 98.26% and *M. humiferrea* OCP showed an identity of 98.07% to the type strain *M. humiferrea* DSM 23265^T^. The genome of the isolate ACPs was most identical to the genomes of E306M (93.24%) and E308F (93.26%), which correspond to a novel, hitherto, unpublished *Moorella* species. Concerning described *Moorella* isolates, the strain ACPs were most identical to *M. mulderi* DSM 14980^T^ (90.64%) and *M. stamsii* DSM 26217^T^ (89.91%). All determined identities fall below the species threshold of 95%, and thereby imply that ACPs represents a novel species in the *Moorella* genus. We are proposing the species name *M. carbonis* for the ACPs isolate.

#### Genome-based metabolic analysis

**Figure 3.**
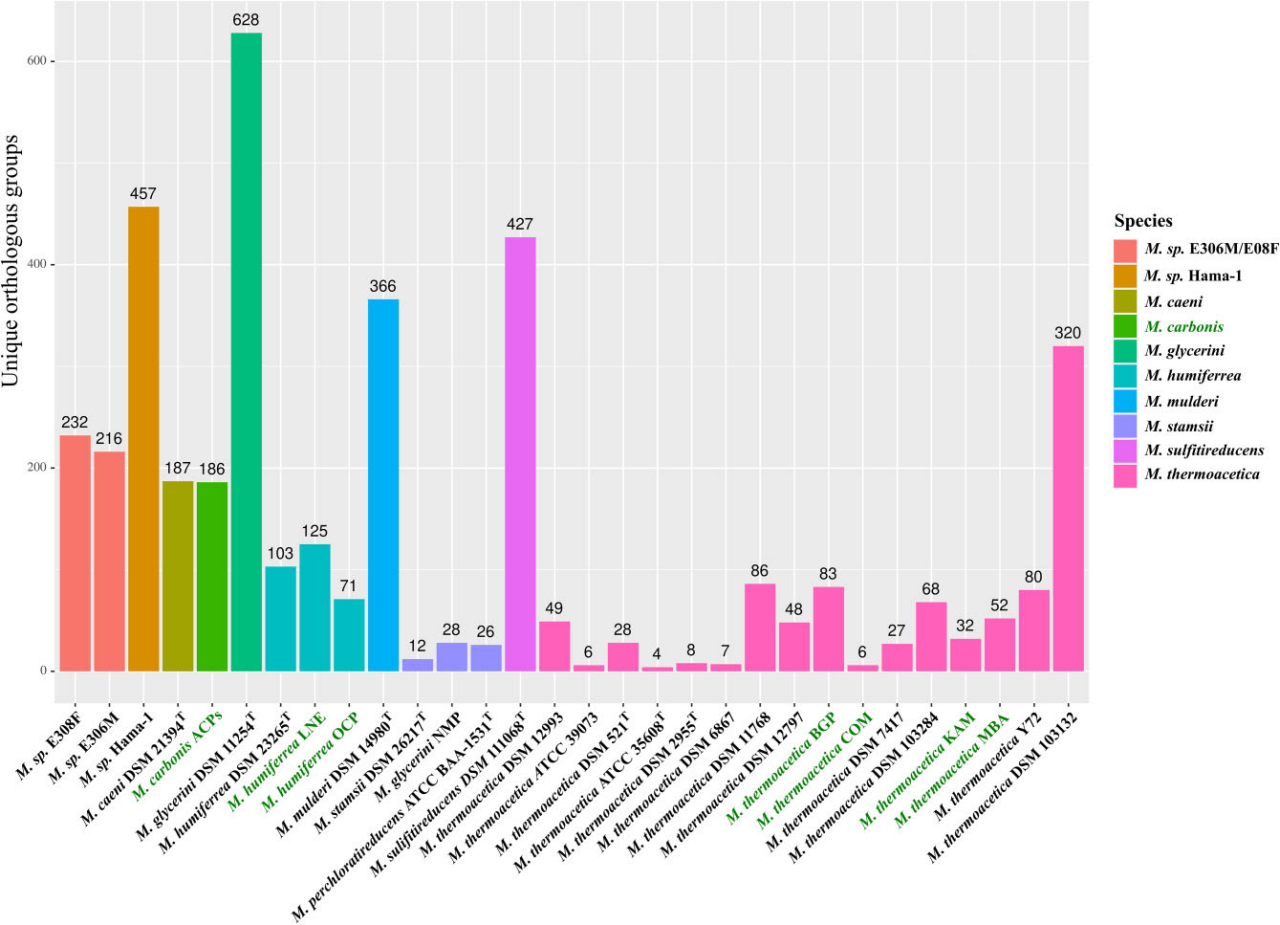
Detected unique OGs in novel and published *Moorella* genomes clustered by species. Novel *Moorella* isolates from this study are highlighted in green and the species colour code is shown on the right.

**Figure 4.**
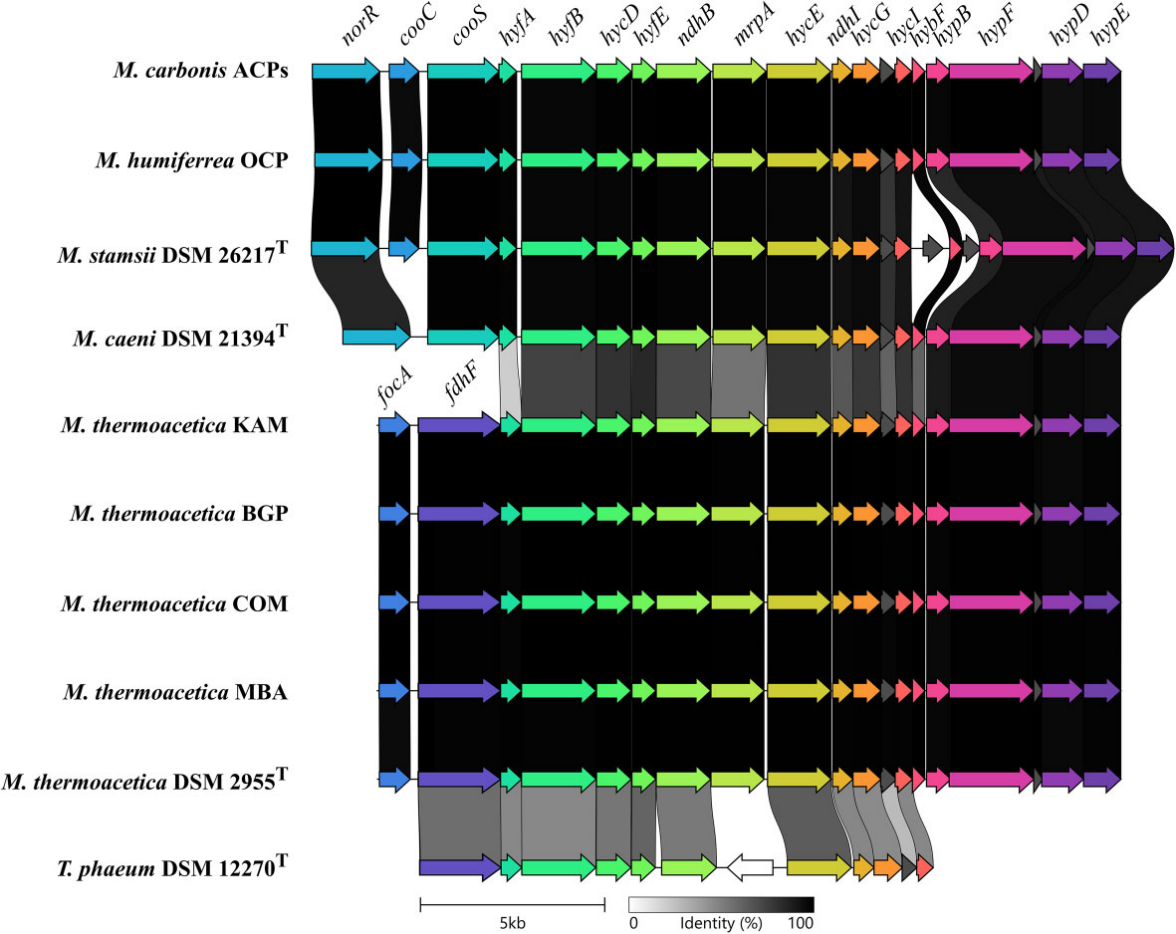
Structures of the different gene cluster encoding the energy-converting hydrogenase complex (Ech1) in *Moorella* strains. The *ech1* gene cluster from the type strains of published *Moorella* species was compared to the *ech1* gene cluster of novel *Moorella* isolates. The Ech1 complex was encoded by the genes: *hyfA*, Hydrogenase-4 component A; *hyfB*, hydrogenase-4-component B; *hycD*, formate hydrogenlyase subunit 4; *hyfE*, hydrogenase-4-component E; *ndhB*, NAD(P)H-quinone oxidoreductase subunit 2; *mrpA*, Na^+^/H^+^ antiporter subunit A; *hycE*, hydrogenase-3-component E; *ndhI*, NAD(P)H-quinone oxidoreductase subunit 1; *hycG*, hydrogenase-3-component G; and *hycI*, hydrogenase 3 maturation protease. The *ech1* gene cluster was followed by hydrogenase maturation factors encoded by the genes: *hybF*, hydrogenase maturation factor HybF; *hypB*, hydrogenase maturation factor HypB; *hypF*, carbamoyltransferase HypF; *hypD*, hydrogenase maturation factor HypD; and *hypE*, carbamoyl dehydratase HypE. Upstream of the *ech1* gene cluster, the strains *M. carbonis* ACPs, *M. humiferrea* OCP, *M. stamsii* DSM 26217^T^, and *M. caeni* DSM 21394^T^ were found to encode: *norR*, anaerobic nitric oxide reductase transcription regulator; *cooC*, carbon monoxide dehydrogenase accessory protein; and *cooS*, carbon monoxide dehydrogenase. In contrast, *M. thermoacetica* strains encoded upstream of the *ech1* gene cluster: *focA*, formate transporter and *fdhF*, formate dehydrogenase H.

After obtaining a phylogenomic classification of the novel *Moorella* isolates, we performed a genome-based functional prediction to assess the differences between the novel isolates and other available *Moorella* strains. Protein sequences from all *Moorella* genomes were clustered to OGs with Proteinortho. The genus *Moorella* formed a pan genome of 9993 OGs, of which 1286 OGs represented the core genome. OGs not present in other *Moorella* genomes were defined as unique OGs and grouped to a representative species based on the previous phylogenomic analysis (Fig. 3). *Moorella glycerini* DSM 11254^T^ (628), *M*. sp. Hama-1 (457), *M. sulfitireducens* DSM 110068^T^ (427), and *M. mulderi* DSM 14980^T^ (366) contained the highest number of unique OGs, followed by the species *Moorella* sp. E308F/E306M (232/216), *M. caeni* DSM 21394^T^ (187), and *M. carbonis* (186). *Moorella humiferrea* DSM 23265^T^ (103) and *M. stamsii* DSM 26217^T^ (12) contained the least number of unique OGs compared to the type strain genomes of other *Moorella* species. The genome of *M. carbonis* contained unique OGs, encoding propanediol/ethanolamine utilization proteins (*pdu* cluster). The structure of the *pdu* cluster in *M. carbonis* was similar to the *pdu* clusters described for *Salmonella typhimurium* and *Acetobacterium woodii* (Fig. S1). In addition to propanediol utilization proteins, this gene cluster has been shown to encode proteins allowing the self-assembly of bacterial microcompartments to protect the cell from the toxic propionaldehyde intermediate during 1,2-propanediol degradation (Bobik et al. 1999, Schuchmann et al. 2015). The presence of a *pdu* gene cluster in acetogens varies from species to species and does not follow phylogenetic patterns. Other examples for acetogens with a *pdu* gene cluster are *Acetonema longum* (Kane and Breznak 1991), *Clostridium carboxidivorans, Clostridium scatologenes*, and *Eubacterium limosum* (Schuchmann et al. 2015). Degradation of rhamnose and fucose derived from plant cell material is the major source of 1,2-propanediol in anaerobic environments (Dank et al. 2021). Other unique OGs of *M. carbonis* encoded a permease for sialic acid, xylulose kinase, carbon monoxide dehydrogenase, phosphopantetheine adenylyltransferase, 1,4-dihydroxy-2-naphthoyl-CoA hydrolase, NADH oxidase, 4-hydroxybutyryl-CoA dehydratase, and subunits HndA/HndD of an NADP-reducing hydrogenase. The *M. humiferrea* isolates OCP and LNE possessed 71 and 125 unique OGs. The latter isolate LNE encoded two unique genes annotated as 1,3-propanediol dehydrogenase and a gene encoding an aldehyde–alcohol dehydrogenase. The *M. thermoacetica* type strains and the other highly similar strains identified in the ANIm analysis contained 4–28 unique OGs, while 6–86 unique OGs were identified in the remaining *M. thermoacetica* genomes. The only execption was *M. thermoacetica* DSM 103132, containing 320 unique OGs. The isolate KAM contained 32 unique OGs, including genes encoding a xanthine permease, putative thiosulfate sulfurtransferase, and glycerol kinase. The isolates BGP, COM, and MBA contained 83, 6, and 52 unique OGs, respectively, of which most were assigned hypothetical functions or did not confer unique metabolic traits. After the identification of unique genomic traits, the genomes of the novel *Moorella* isolates were analyzed with regard to genes involved in acetogenesis. Every genome of the *Moorella* isolates encoded a trimeric electron-bifurcating hydrogenase (*hyd*ABC) (Wang et al. 2013) and a dimeric electron-bifurcating transhydrogenase (*nfn*AB), linking the redox pool of NADH and reduced ferredoxin to the cellular redox pool of NADPH (Huang et al. 2012). Recently, a hexameric hydrogenase (HydABCDEF) was purified from *M. thermoacetica* DSM 521^T^, catalyzing H_2_-dependent reduction of NADP (Rosenbaum and Müller 2024). The respective gene structure was identified in all genomes of novel *M. thermoacetica* isolates, but was absent in the ACPs, OCP, LNE, and the *M. humiferrea* type strain DSM 23265^T^ genomes. Furthermore, each genome contained one *mtaABC* gene cluster encoding a methanol methyltransferase and an *mtvABC* gene set, encoding a methyltransferase transferring methyl groups from methoxylated monoaromates. This observation differs from the reported presence of duplicated *mtaABC* and *mtvABC* gene sets in the genome of strain *M. thermoacetica* ATCC 39073, participating in the transfer of methyl groups from methoxylated aromatic compounds (Naidu and Ragsdale 2001, Das et al. 2007, Pierce et al. 2008). All isolates encoded a complete Ech2 complex, but differed in the presence and synteny of the Ech1 complex (Fig. 4). The Ech1 complex was missing in *M. humiferrea* DSM 23265^T^ and *M. humiferrea* LNE. However, the genome of *M. humiferrea* OCP contained all genes of the Ech1 complex followed by hydrogenase maturation factors. Upstream of the *ech1* gene cluster, a transcriptional regulator, a carbon monoxide dehydrogenase maturation factor, and a monofunctional carbon monoxide dehydrogenase were encoded. This gene structure was also identified in the *M. carbonis* ACPs, *M. stamsii* DSM 26271^T^, and *M. caeni* DSM 21394^T^ genomes. The latter was, however, missing the *cooC* gene upstream of the carbon monoxide dehydrogenase gene *cooS*. Both *M. stamsii* DSM 26217^T^ (Alves et al. 2013) and *M. caeni* DSM 21394^T^ (Vecchini Santaella et al. 2023) were described to grow on CO by employing the water–gas shift reaction producing H_2_ and CO_2_. Encoding genes for a monofunctional carbon monoxide dehydrogenase upstream of the *ech1* gene cluster appears to be a characteristic of hydrogenogenic carboxydotrophic *Moorella* strains. This indicated that the isolates *M. humiferrea* OCP and *M. carbonis* ACPs also grow on CO by performing the water–gas shift reaction. A monofunctional carbon monoxide dehydrogenase was present adjacent to the *ech1* gene cluster in *A. autotrophica* DSM 108286^T^ and was shown to be essential for growth on CO in *T. kivui* DSM 2030^T^ (Jain et al. 2022). However, in *T. kivui* the monofunctional carbon monoxide dehydrogenase is located in proximity of the gene cluster for the Ech2 complex, which was shown to be the essential ferredoxin-oxidizing hydrogenase for growth and conversion of CO in this strain (Baum et al. 2024). The isolates KAM, MBA, BGP, and COM contained the full set of genes for the Ech1 complex and the adjacent hydrogenase maturation factors described for *M. thermoacetica* type strains. Similar to the genome of *T. phaeum* DSM 12270^T^, a formate dehydrogenase is encoded upstream of the *ech1* gene cluster. However, in genomes of *M. thermoacetica* strains, an additional formate transporter (FocA) is encoded prior to the formate dehydrogenase. The selenocysteine-containing formate dehydrogenase upstream of the *ech1* gene cluster is missing entirely in the isolates ACPs, LNE, OCP, and *M. stamsii* DSM 26217^T^. However, all isolates encoded another formate dehydrogenase. The isolates KAM, BGP, COM, and MBA encode a selenocysteine-containing formate dehydrogenase consisting of an alpha and beta subunit as found in the *M. thermoacetica* type strains and the *M. caeni* type strain. The isolates ACPs, LNE, OCP, and the type strains of the species *M. glycerini, M. sulfitireducens, M. humiferrea*, and *M. mulderi* only encode the alpha subunit of the formate dehydrogenase. Prior to the alpha subunit of the formate dehydrogenase, genes were identified encoding proteins with similarity to the *hydC* and *hydB* gene of the trimeric electron-bifurcating HydABC hydrogenase and the hexameric NADP-reducing HydABCDEF complex of the *M. thermoacetica* strains. In all isolate genomes the genes for the methyl and carbonyl branch of the WLP were encoded in an ~20 kb gene cluster, also containing *hdrA, hdrB, hdrC*, and *mvhD* upstream of *metV* and *metF*. Furthermore, all contained *pduL* genes with phosphotransacetylase function and an acetate kinase. The putatively electron-bifurcating FixABC complex was identified in all isolate genomes (Garcia Costas et al. 2017). With the exception of the ACPs isolate, all genomes encoded a ferredoxin-like protein (FixX) and the cytochrome b (Bfr) downstream of the *fixABC* gene clusters. The ACPs isolate genome encoded two *fixABC* gene clusters; however, *fixX* and *bfr* were encoded by another genomic region and not adjacently. One cluster of the *fixABC* genes was found in synteny with a thiolase (Thl), a 3-hydroxybutyryl-CoA dehydrogenase (Hbd), an acyl-CoA dehydrogenase (Bcd) upstream of the *fixABC* genes, followed by a phosphopantetheine adenylyltransferase (CoaD) and a crotonase (Crt). Other *Moorella* species genomes, which contained a complete gene set for hypothetical butyrate production, were *M. stamsii* DSM 26217^T^, *M. perchloratireducens* ATCC BAA-1531^T^, *M. glycerini* NMP, and *M. sulfitireducens* DSM 111068^T^. However, only the ACPs isolate showed all of these genes organized in one cluster (Fig. S2). A similar gene cluster was found in the *M. sulfitireducens* DSM 111068^T^ genome, but lacking the *hbd* and *crt* in synteny. Both genes were encoded in two other genomic regions in *M. sulfitireducens* each containing one copy of *hbd* and *crt*. The phosphotransacetylase reaction in *Moorella* is catalyzed by the phosphate propanoyltransferase (PduL) and was encoded in the ACPs isolate by four gene copies (Breitkopf et al. 2016). Together with two genes annotated as acetate kinase (*ack*) and one gene annotated as butyrate kinase (*buk*), the ACPs isolate genome harbored a full set of genes coding for proteins required for the theoretical production of butyrate from acetyl-CoA. CO-dependent butyrate production has been described for the acetogen *Eubacterium callanderi* KIST612 and ethanol/acetate-dependent butyrate production for *Clostridium kluyveri* both employing a complex of Bcd with an electron-bifurcating flavoprotein (Etf/Bcd complex) (Li et al. 2008, Jeong et al. 2015). The *etfA/B* genes of these butyrate-producing strains were similar to the *fixA/B* genes in *M. sulfitireducens* and *M. carbonis* strains, which additionally encoded the genes *fixC* and *coaD* adjacently.

#### Prophage activity screening

**Figure 5.**
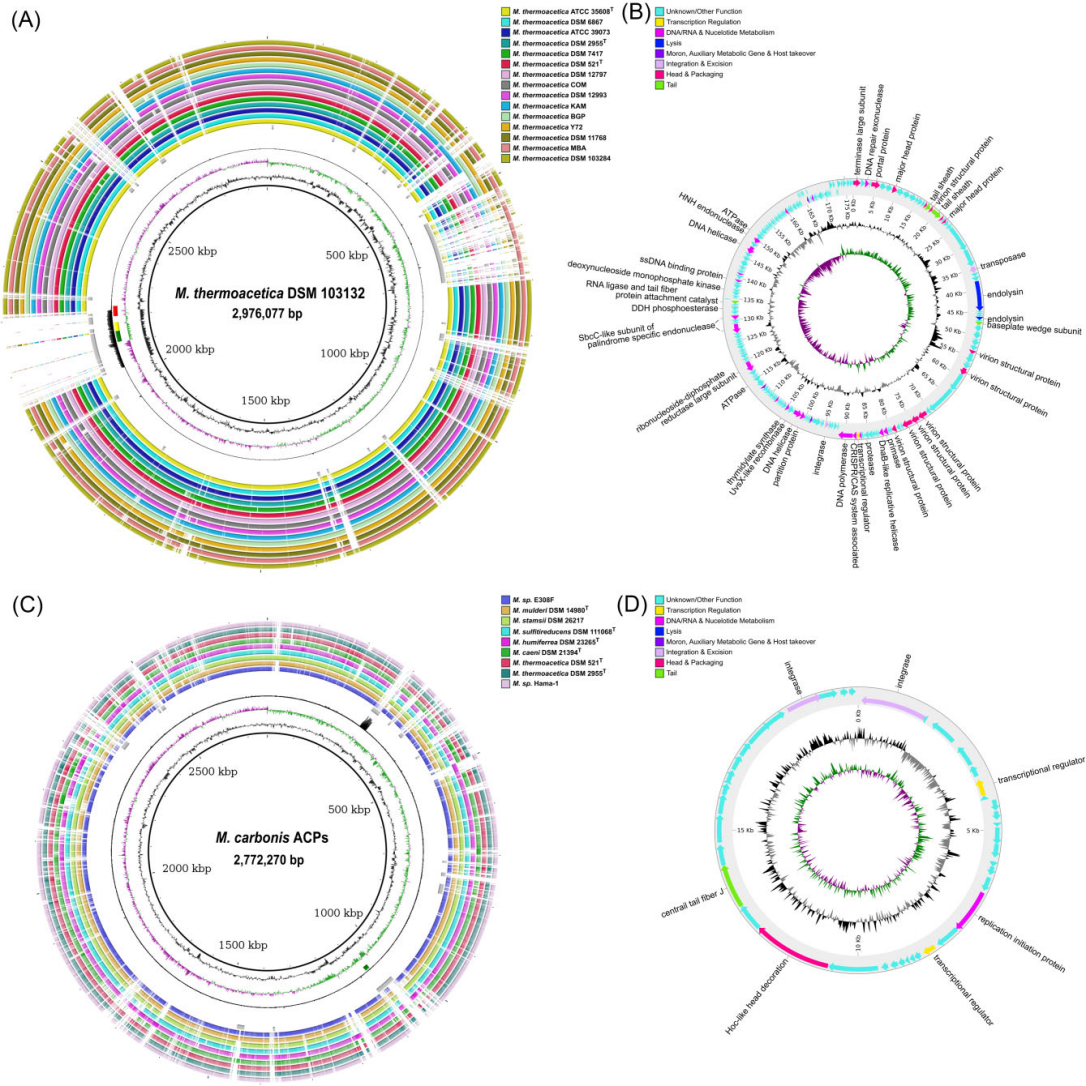
Prophage activity screening of the strains *M. thermoacetica* DSM 103132 and *M. carbonis* ACPs. Visualization of the host genome in comparison to closely related genomes of *Moorell*a strains (A and C) with BLASTn. The order of the inner rings is: host reference genome (black), GC content of host genome (black), positive (purple), and negative (green) GC skew, prophage prediction by PHASTEST (red, incomplete; yellow, questionable; and green, intact), coverage graph of sequenced DNA from isolated phage particles (black), genomic islands prediction by IslandViewer4 (grey), and remaining rings show the BLASTn comparison with other *M. thermoacetica* strains (A) or other *Moorella* species (C) (colour code in the legend at the top right). Inferred thermophage genome sequences from active prophage regions detected in *M. thermoacetica* DSM 103132 (B) and *M. carbonis* ACPs (D). The colour code for the function of annotated genes is shown at the top left.

Because severe growth inhibition can be caused by active prophages, we performed a prophage activity screening for all of our isolates and the strains *M. thermoacetica* DSM 521^T^, DSM 2955^T^ DSM 103132, and *M. humiferrea* DSM 23265^T^. Of all 11 analyzed *Moorella* strains, only two were identified to contain active prophages (Fig. 5). *Moorella thermoacetica* DSM 103132 was predicted to contain two large genomic islands with the size of ~200 kb obtained by horizontal gene transfer. This also explains the unusual amount of unique gene clusters of the DSM 103132 genome detected during the genomic characterization in contrast to other *M. thermoacetica* strains. The prophage activity screening showed an active region with the size of 176 kb flanked by tRNA genes on both sides. The active region aligned with one of the large genomic islands predicted by Island Viewer 4 and three prophage regions (intact, questionable, and incomplete) predicted by PHASTEST (Fig. 5A). Pharokka annotated multiple phage-related genes, such as terminase, exonuclease, endolysin, primase, helicase, integrase, protease, and peptidase. Several phage structure proteins were annotated including genes for tail sheath, baseplate wedge, and tail fiber protein attachment catalyst (Fig. 5B). Genes encoding proteins for a tail with a baseplate indicate an affiliation of the detected bacteriophage to the *Myoviridae* family. The *M. carbonis* DSM 116161^T^ genome showed an active prophage region with the size of 20 kb, which matched to a predicted genomic island, but not to the predicted intact prophage region by PHASTEST (Fig. 5C). Pharokka annotated genes encoding two integrases, two transcriptional regulators, a replication initiation protein, a Hoc-like head decoration protein, and a central tail fiber J protein (Fig. 5D). The latter gene indicates a tailed bacteriophage, and thereby an association to the order *Caudovirales*. Bacteriophages of thermophilic bacteria are of particular interest due to their thermostable proteins and enzymes. Active prophages of bacteria from the genus *Moorella* have not yet been described to the best of our knowledge. The vast majority of described thermophages is associated to the bacterial family of *Bacillaceae* (Łubkowska et al. 2021).

### Physiological characterization

The *M. humiferrea* isolates LNE and OCP were not characterized in detail as they exhibited insufficient heterotrophic growth in M-medium in comparison to the other isolates. The *M. humiferrea* isolates grew to an OD_600_ of 0.01 (LNE) and 0.03 (OCP) after 3 days of incubation at 60°C in M-medium with both fructose (30 mM) and dl-lactate (30 mM) as substrates. Growth optima for all characterized isolates are summarized in Table 2. The analyzed *Moorella* isolates grew at temperatures between 45°C and 70°C. Most isolates showed a temperature optimum at 60°C, only the BGP isolate grew best at 65°C. The *Moorella* isolates were not able to grow at NaCl concentrations higher than 1%. The isolates KAM and MBA were most susceptible to increasing NaCl concentrations and grew optimally in medium in which NaCl addition was omitted. All other strains showed a NaCl optimum at 0.2%. Growth was detected at pH values between 4.5 and 9.5. Strain KAM grew optimally at pH 7 and the isolates MBA and ACPs showed optimal growth at pH 7.5. The BGP and COM isolate had the highest optimum at pH 8. All isolates stained Gram positive.

**Table 2.** Physiological characterization of novel *Moorella* isolates.

	*M. thermoacetica* KAM	*M. thermoacetica* BGP	*M. thermoacetica* COM	*M. thermoacetica* MBA	*M. carbonis* ACPs^T^
Gram staining	Positive	Positive	Positive	Positive	Positive
Temperature—optimum	60°C	65°C	60°C	60°C	60°C
Temperature—range	50°C–70°C	45°C–70°C	45°C–65°C	55°C–65°C	55°C–70°C
NaCl—optimum	0%	0.2%	0.2%	0%	0.2%
NaCl—range	0%–0.8%	0%–1%	0%–1%	0%–1%	0%–1%
pH—optimum	7	8	8	7.5	7.5
pH—range	5–9.5	4.5–9.5	5–9.5	5–9	4.5–9

The results from the substrate growth tests are summarized in Table S3. All novel *M. thermoacetica* isolates showed the best growth on fructose and were able to utilize glucose, *myo*-inositol, sucrose, raffinose, formate, lactate, vanillate, syringate, and methanol. The KAM isolate was the only isolate growing on glucuronic acid and glycerol, utilization of the latter substrate does match to the unique glycerol kinase-encoding gene identified during genomic comparison. Fermentation of glycerol has been described for *M. glycerini* (Slobodkin et al. 1997), *M. sulifitreducens* (Slobodkina et al. 2022), and *M. humiferrea* (Nepomnyashchaya et al. 2012) (with AQDS as electron acceptor), but to our knowledge not for an *M. thermoacetica* strain. Strain BGP was the only *M. thermoacetica* isolate analyzed showing growth with xylose and *n*-butanol; however, utilization of both substrates is described as a physiological feature of *M. thermoacetica* (Drake and Daniel 2004). Strain COM was the only isolate showing growth on sorbitol and mannitol. Both sugar alcohols are abundantly available in biomass, and thus matched to the compost environment it was isolated from (Pleyerová et al. 2022). Best growth on methanol was achieved by the isolates COM and MBA, which were both isolated from compost samples. In the compost environment, methoxylated compounds are abundantly available from the degradation of lignin (Weng et al. 2021), which may have improved one-carbon substrate utilization of the isolates COM and MBA. The ACPs isolate was the only strain able to grow with ribose, rhamnose, and 1,2-propanediol. The latter two substrates can be found in plant-degrading environments, as rhamnose is abundantly found in plant cell walls and the product of anaerobic rhamnose degradation is 1,2-propanediol (Dank et al. 2021). The utilization of 1,2-propanediol was in line with the identification of the for *Moorella* strains, hitherto unique *pdu* gene cluster identified in *M. carbonis*. Nitrate and DMSO were utilized as electron acceptors when *M. carbonis* was supplied with fructose and glycerol as electron donors. Thiosulfate, sulfite, sulfate, perchlorate, fumarate, AQDS, and nitrite were not utilized as electron acceptors. Nitrate reduction was also described for the species *M. stamsii* (Alves et al. 2013), *M. thermoacetica* (Drake and Daniel 2004), and *M. humiferrea* (Nepomnyashchaya et al. 2012). DMSO reduction was described for *M. thermoacetica* and not investigated in other *Moorella* species (Rosenbaum et al. 2022). The cellular fatty acid analysis of *M. carbonis* ACPs is shown in Table S4 and the results were compared to the cellular fatty acid profiles reported for *M. stamsii* DSM 26217^T^, *M. glycerini* DSM 11254^T^, *M. humiferrea* DSM 23265^T^ (Alves et al. 2013), and *M. caeni* DSM 21394^T^ (Vecchini Santaella et al. 2023). The major fatty acids detected were Iso-C_15:0_ (40.1%), Iso-C_15:0_ DMA (23.1%), and Iso-C_17:0_ DMA (8.9%). In comparison to the cellular fatty acid profiles of *M. stamsii, M. glycerini*, and *M. humiferrea*, strain ACPs contained higher proportions of Iso-C_15:0_. In contrast, the profile of *M. caeni* showed higher proportions of Iso-C_15:0_. The isolate ACPs contained higher proportions of C_14:0_ and Iso-C_15:0_ DMA than detected in every other profile. Furthermore, strain ACPs possessed considerably lower proportions of C_16:0_, Iso-C_17:0_, and C_18:0_. The fatty acid Iso-C_17:0_ DMA was only detected in *M. carbonis*. In conclusion, the cellular fatty acid analysis further supported the classification of strain ACPs as a novel species from the genus *Moorella*.

#### Autotrophic growth

**Figure 6.**
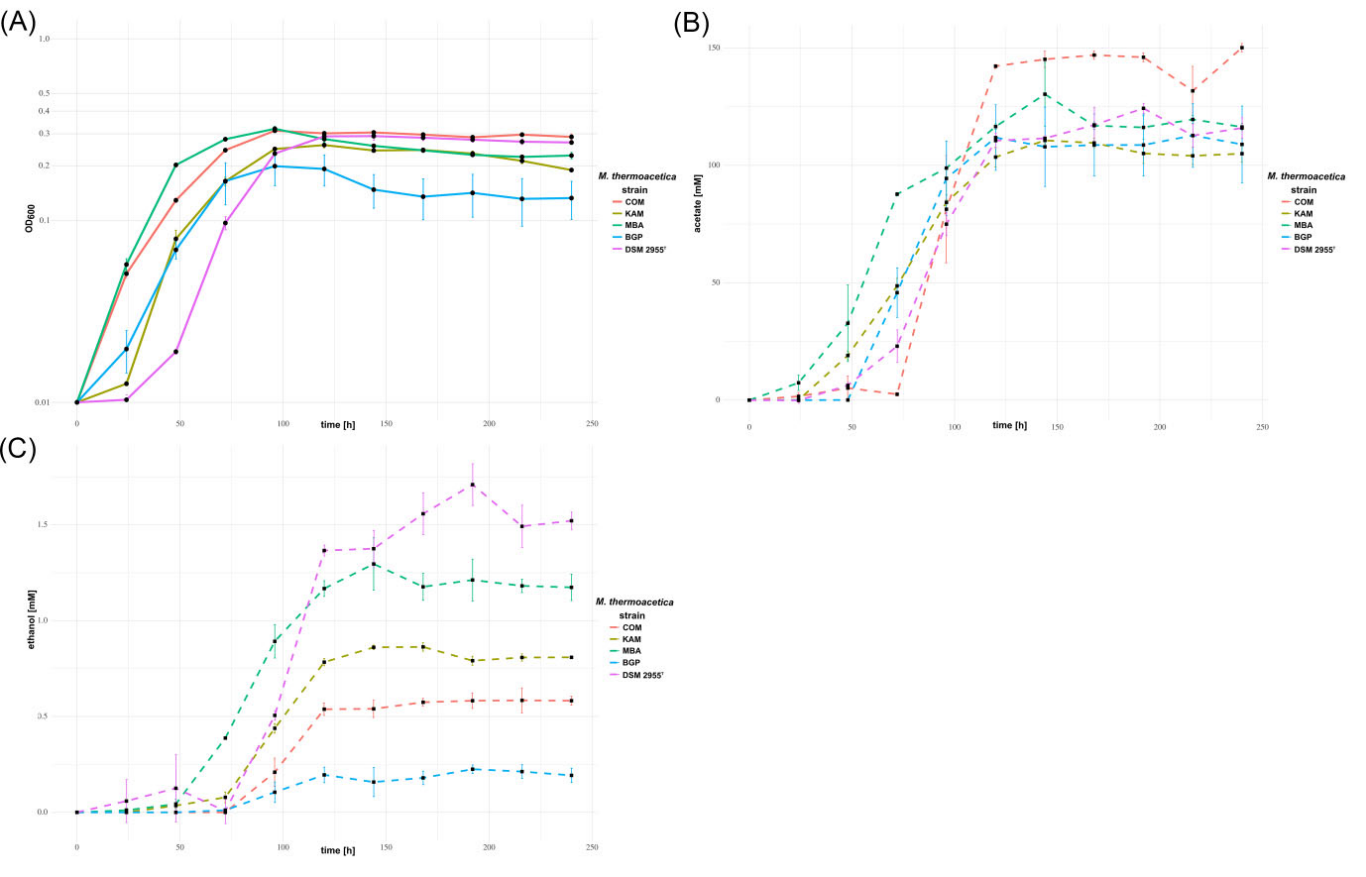
Growth profiles of *M. thermoacetica* strains with H_2_ + CO_2_ and detected fermentation products. Growth on H_2_ + CO_2_ of the novel *M. thermoacetica* isolates KAM, BGP, COM, and MBA with DSM 2955^T^ as reference strain plotted as mean OD_600_ of triplicate cultures on a logarithmic scale over the course of 240 h (A), with the corresponding GC measurements of mean acetate production (B) and mean ethanol production (C) in mM.

Substrates for autotrophic growth of the novel *Moorella* isolates and the *M. humiferrea* type strain are summarized in Table 3. The *M. humiferrea* isolate LNE grew with H_2_ + CO_2_ and CO, producing acetate as the sole fermentation product. The *M. humiferrea* DSM 23265^T^ type strain utilized neither H_2_ + CO_2_ nor CO, in line with the type strain description and the growth experiments performed by Alves and colleagues (Nepomnyashchaya et al. 2012, Alves et al. 2013). As shown in our genome-based metabolic analysis, the *M. humiferrea* LNE strain lacks the Ech1 complex and therefore relies only on the Ech2 complex to build a chemiosmotic gradient during autotrophic growth. The strains *M. carbonis* ACPs and *M. humiferrea* OCP both isolated from charcoal burning pile environments did not utilize H_2_ + CO_2_ but transformed CO to H_2_ + CO_2_. This carboxydotrophic hydrogenogenic metabolism was also described for *M. stamsii* DSM 26217^T^ (Alves et al. 2013) and *M. caeni* DSM 21394^T^ (Vecchini Santaella et al. 2023). The genomes of all four of these strains encoded a monofunctional carbon monoxide dehydrogenase in synteny with genes encoding the Ech1 complex, thereby confirming this gene structure to be a characteristic of carboxydotrophic hydrogenogenic *Moorella* strains. As both carboxydotrophic hydrogenogens in this study were isolated from the charcoal burning pile environments, this metabolism hypothetically serves as an adaptation of acetogenic bacteria to cope with high concentrations of the toxic substrate CO. This putatively leads to a focus on the conversion of CO for rapid removal from the cell and to a loss of the ability to utilize the H_2_ + CO_2_ produced by the water–gas shift reaction. We did not detect butyrate production by *M. carbonis* ACPs grown on fructose or CO. The *M. thermoacetica* isolates KAM, BGP, COM, and MBA, and the type strain DSM 2955^T^ utilized both H_2_ + CO_2_ and CO, which is in line with the physiological description of *M. thermoacetica* strains (Daniel et al. 1990). We performed detailed analysis for the growth of these five strains on H_2_ + CO_2_ also assessing the produced fermentation products (Fig. 6A–C). The fastest growing strains were MBA and COM, which also achieved the highest end ODs. The DSM 2955^T^ strain showed a longer lag phase than the other isolates, but finally achieved a similar end OD as the best growing strains MBA and COM. The KAM and BGP isolates grew slower and achieved a lower end OD than the other isolates. COM produced the highest amount of acetate from H_2_ + CO_2_, also in comparison to strains achieving a similar end OD. We detected low concentrations of ethanol in the culture supernatants of all five *M. thermoacetica* strains after 96 h of incubation. The highest amounts of ethanol were detected in cultures of DSM 2955^T^, MBA, and KAM. The production of small amounts of ethanol from H_2_ + CO_2_ has been described for the strains HUCC22-1 and Y72, but our results indicate that the production of ethanol could be a general trait of *M. thermoacetica* strains (Sakai et al. 2005, Kimura et al. 2016). The enzymes involved in ethanol formation from acetyl-CoA in strain HUC22-1 were an aldehyde dehydrogenase Aldh and the alcohol dehydrogenase AdhA (Inokuma et al. 2007). Indeed, in the genomes of all five *M. thermoacetica* strains, orthologues of Aldh (MTKAM_04420/18780, MTBGP_20370, MTCOM_17670, MTMBA_20270, and MOTHA_18730) and AdhA (MTKAM_20210, MTBGP_5400/21960, MTCOM_19130, MTMBA_21660, and MOTHA_20270) were detected. Other alcohol dehydrogenases detected were AdhB (MTKAM_10310, MTBGP_12010, MTCOM_9650, MTMBA_12080, and MOTHA_10720), AdhC (MTKAM_23920, MTMBA_25190, and MOTHA_24090), and a putative zinc-type alcohol dehydrogenase-like protein YdjJ (MTCOM_21580). Nevertheless, all five *M. thermoacetica* strains also encoded at least three copies of an aldehyde ferredoxin oxidoreductase (AOR). This enzyme has originally been described from *M. thermoacetica* (White et al. 1989). In conjunction with an Adh, AOR has been demonstrated to be involved in ethanol formation from sugars by direct ferredoxin-dependent reduction of acetate in the hyperthermophilic archaeon *Pyrococcus furiosus* (Basen et al. 2014), and from synthesis gas in the acetogen *Clostridium autoethanogenum* (Liew et al. 2017). For *M. thermoacetica*, ethanol production from acetyl-CoA could be increased by deletion of *pduL* genes and occurred during growth on H_2_ + CO_2_ and CO in genetically modified strains of *M. thermoacetica* ATCC 39073 (Takemura et al. 2021).

**Table 3.** Autotrophic growth of analyzed *Moorella* strains in M-medium (0.5 g/l yeast extract) on gaseous substrates and corresponding fermentation products.

Substrate	*M. thermoacetica* KAM	*M. thermoacetica* BGP	*M. thermoacetica* COM	*M. thermoacetica* MBA	*M. carbonis* ACPs	*M. humiferrea* DSM 23265^T^	*M. humiferrea* LNE	*M. humiferrea* OCP
H_2_ + CO_2_	+ acetate ethanol	+ acetate ethanol	+ acetate ethanol	+ acetate ethanol	−	−	+ acetate	−
CO	+ n.d.	+ n.d.	+ n.d.	+ n.d.	+ H_2_ + CO_2_	−	+ acetate	+ H_2_ + CO_2_

+, positive; −, negative; and n.d., not determined.

### Description of *M. carbonis* sp. nov.

*Moorella carbonis* (car.bo’nis. L. gen. masc. n. *carbonis*, of coal, of charcoal). Cells are Gram-positive rods and produce terminal endospores. Growth occurs only under strict anaerobic conditions and is dependent on the addition of yeast extract. Growth ranges from 55°C to 70°C (optimum 60°C), at pH 4.5–9 (optimum 7.5) and NaCl concentrations of 0%–1% (optimum 0.2%). Best growth occurs with xylose and fructose. Furthermore, the substrates ribose, galactose, glucose, mannose, rhamnose, *myo*-inositol, sucrose, raffinose, melezitose, formate, pyruvate, lactate, vanillate, syringate, DMG, betaine, methanol, and 1,2-propanediol are utilized. CO is converted to H_2_ and CO_2_ as the major product. Growth does not occur on H_2_ + CO_2_, arabinose, glucuronic acid, glycerol, sorbitol, mannitol, cellobiose, trehalose, lactose, maltose, melibiose, dextran, dextrin, starch, oxalate, malate, citrate, ethanol, *n*-propanol, and *n*-butanol. Nitrate and DMSO are used as alternative terminal electron acceptors during growth on fructose and glycerol. The predominant cellular fatty acids are iso-C_15:0_ (40.1%), iso-C_15:0_ DMA (23.1%), and iso-C_17:0_ DMA (8.9%). The type strain genome is 2.8 Mbp in size, has a GC content of 55%, and encodes a complete gene cluster for the utilization of 1,2-propanediol-degrading microcompartments. The type strain ACPs was isolated from the covering soil of an active charcoal burning pile, Hasselfelde, Germany. The GenBank/EMBL/DDBJ accession number of the 16S rRNA gene is OR576718. The type strain of *M. carbonis* is ACPs^T^ and was deposited under the identifiers DSM 116161^T^ and CCOS 2103^T^.

## Conclusions

We isolated seven novel *Moorella* strains from different environments by conducting autotrophic and heterotrophic enrichments. The most successful substrates for cultivation of *Moorella* strains were H_2_ + CO_2_, vanillate, and methanol. High-quality genomes were obtained for all isolates and a genome-based comparison to available *Moorella* genomes yielded, besides a phylogenomic classification also, the identification of potential unique metabolic traits. The two novel isolates OCP and ACPs from charcoal burning pile soil samples were shown to be carboxydotrophic hydrogenogens and shared a characteristic gene structure encoding a monofunctional CO dehydrogenase in synteny to the *ech1* gene cluster. This structure was also identified in the carboxydotrophic hydrogenogens *M. stamsii* and *M. caeni*. The isolate ACPs was proposed as the type strain of a novel *Moorella* species with the name *M. carbonis*. With the OCP isolate and the LNE isolate enriched from river sediment, we obtained two *M. humiferrea* strains growing autotrophically with gaseous substrates, in contrast to the *M. humiferrea* type strain. While the OCP isolate was a carboxydotrophic hydrogenogenic strain, the LNE isolate was shown to ferment H_2_ + CO_2_ and CO to acetate. The other four isolates KAM, BGP, COM, and MBA were identified as novel strains of *M. thermoacetica*. Both of the latter isolates grew considerably better on methanol than other *Moorella* strains. All four novel *M. thermoacetica* strains were shown to produce small amounts of ethanol from H_2_ + CO_2_. As this was also demonstrated for the *M. thermoacetica* DSM 2955^T^ type strain, it is hypothesized that ethanol production from H_2_ + CO_2_ is a common trait of most *M. thermoacetica* strains, hitherto often described as homoacetogens. This does imply that acetogenesis from syngas mixtures may be switched to ethanologenesis, by disabling the phosphotransferase PduL without requiring the introduction of external genes for ethanol production in *M. thermoacetica* strains. Furthermore, we detected prophage activity in *M. thermoacetica* DSM 103132 and *M. carbonis* DSM 116161^T^, which provided first insights into thermophages associated with the genus *Moorella*.

## Supplementary Material

fiae109_Supplemental_Files

## Data Availability

The GenBank/EMBL/DDBJ/SRA accession numbers of genome and phage sequencing data are listed in Table S2. Raw sequencing data of the bacterial community analysis of enrichment cultures are available under the BioProject PRJNA1019916, BioSample SAMN37505147–SAMN37505208 and SRA SRR26145583–SRR26145525.
