# Supplementary material for: Isolation and characterization of novel acetogenic Moorella strains for employment as potential thermophilic biocatalysts
Source: FEMS Microbiol Ecol. 2024 Aug 8;100(9):fiae109. doi: 10.1093/femsec/fiae109 (PMC11328732; doi:10.1093/femsec/fiae109)
Supplement: fiae109_Supplemental_Files [file fiae109_supplemental_files.zip › Supplementary data Figure legends.docx]

**Fig. S1** Gene structure of the propanediol utilization gene cluster detected in the ACPs isolate in comparison to *S. typhimurium* DSM 17058^T^ and *A. woodii* DSM 1030^T^. The following gene abbreviatons were used: *pduA*, BMC shell protein PduA; BMC shell protein PduB; *pduC*, propanediol dehydratase large subunit; *pduD*, propanediol dehydratase medium subunit; *pduE*, propanediol dehydratase small subunit; *pduG*, propanediol dehydratase-reactivating factor large subunit; *pduH*, propanediol dehydratase-reactivating factor small subunit; *pduJ*, BMC shell protein PduJ; *pduL*, phosphate propanoyltransferase; *pduM*, BMC shell protein PduM; *pduN*, BMC shell protein PduN; *pduO*, corrinoid adenosyltransferase PduO, *pduP*, propanal dehydrogenase; *pduQ*, 1-propanol dehydrogenase; *pduS*, cobalamin reductase; *pduT*, BMC shell protein PduT; *pduU*, BMC shell protein PduU; *pduV*, propanediol utilization protein PduV; *pduW*, propionate kinase; *pduX*, L-threonine kinase, *buk*, butyrate kinase; *coaBC*, **coenzyme A biosynthesis bifunctional protein CoaBC. *M carbonis* and *A. woodii* both additionally encoded a protein of unknown function with 8 transmembrane helices (8 TMH membrane protein).**

**Fig. S2** Gene cluster for butyrate production in *C. kluyveri*, *E. limosum* and *E. callanderi* in comparison to a similar gene cluster identified in *M. carbonis* and *M. sulfitireducens*. The following genes were identified: *thl*, acetyl-CoA acetyltransferase; *bcd*, butyryl-CoA dehydrogenase; *etfB/fixA*; electron transfer flavoprotein subunit beta; *etfA/fixB*, electron transfer flavoprotein subunit alpha; *fixC*, electron transfer flavoprotein-quinone oxidoreductase; *coaD*, phosphopantetheine adenylyltransferase; *hcd*, 3-hydroxyacyl-CoA dehydrogenase; *crt*, 3-hydroxybutyryl-CoA dehydratase.
