# Supplementary material for: Isolation and characterization of novel acetogenic Moorella strains for employment as potential thermophilic biocatalysts
Source: FEMS Microbiol Ecol. 2024 Aug 8;100(9):fiae109. doi: 10.1093/femsec/fiae109 (PMC11328732; doi:10.1093/femsec/fiae109)
Supplement: fiae109_Supplemental_Files [file fiae109_supplemental_files.zip › Supplementary data Table S1.docx]

Table S1: Accession numbers of analysed *Moorella* reference genomes

| Genome | Assembly accession (GenBank/GOLD) |
| --- | --- |
| *M. sulfitireducens* DSM 111068^T^ | GCA_024733845.1 |
| *M*. 68 (MAG) | GCA_014360145.1 |
| *M*. UBA40 (MAG) | GCA_002383405.1 |
| *M*. SpSt-1133 (MAG) | GCA_011375465.1 |
| *M*. 60_41 (MAG) | GCA_001508035.1 |
| *M*. *sp*. Hama-1 | GCA_023734095.1 |
| *M*. DJKA99_ANTF-F21_bin24 (MAG) | GCA_030445735.1 |
| *M. humiferrea* Bu11 (MAG) | GCA_014896575.1 |
| *M*. *sp*. E306M | GCA_006538385.1 |
| *M*. *sp*. E308F | GCA_006538365.1 |
| *M*. DJKA48_NP-F35_bin7 (MAG) | GCA_030154285.1 |
| *M*. DJKA123_ANTF-F21_bin2 (MAG) | GCA_030156605.1 |
| *M. stamsii* DSM 26217^T^ | GCA_002995805.1 |
| *M. glycerini* NMP | GCA_001373375.1 |
| *M. perchloratireducens* ATCC BAA-1531^T^ | Gp0011525 (GOLD Sequencing Project ID) |
| *M. mulderi* DSM 14980^T^ | GCA_001594015.1 |
| *M. glycerini* DSM 11254^T^ | GCA_009735625.1 |
| *M. caeni* DSM 21394^T^ | GCA_001875325.1 |
| *M*. DJKA89_NP-J24_bin25 (MAG) | GCA_030446125.1 |
| *M. thermoacetica* DSM 12797 | GCA_001874085.1 |
| *M. thermoacetica* DSM 11768 | GCA_001874065.1 |
| *M. thermoacetica* DSM 7417 | GCA_001875265.1 |
| *M. thermoacetica* DSM 103284 | GCA_001874605.1 |
| *M. thermoacetica* DSM 103132 | GCA_001729945.1 |
| *M. thermoacetica* DSM 12993 | GCA_001875245.1 |
| *M. thermoacetica* Y72 | GCA_000576385.2 |
| *M. thermoacetica* ATCC 35608^T^ | GCA_008121885.1 |
| *M. thermoacetica* DSM 6867 | GCA_001875285.1 |
| *M. thermoacetica* DSM 2955^T^ | GCA_001267435.1 |
| *M. thermoacetica* ATCC 39073 | GCA_000013105.1 |
| *M. thermoacetica* DSM 521^T^ | GCA_001267405.1 |
