# Supplementary material for: Isolation and characterization of novel acetogenic Moorella strains for employment as potential thermophilic biocatalysts
Source: FEMS Microbiol Ecol. 2024 Aug 8;100(9):fiae109. doi: 10.1093/femsec/fiae109 (PMC11328732; doi:10.1093/femsec/fiae109)
Supplement: fiae109_Supplemental_Files [file fiae109_supplemental_files.zip › Supplementary data Table S2.docx]

Table S2: Genome features and accession numbers of sequenced or analysed *Moorella* strains

|  | *M. thermoacetica* KAM | *M. thermoacetica* BGP | *M. thermoacetica* COM | *M. thermoacetica* MBA | *M. carbonis* ACPs | *M. humiferrea* 64-FGQ^T^ | *M. humiferrea* OCP | *M. humiferrea* LNE | *M. thermoacetica* DSM 1974-HH |
| --- | --- | --- | --- | --- | --- | --- | --- | --- | --- |
| Culture collection deposit | - | - | - | - | DSM 116161^T^  CCOS 2103^T^ | DSM 23265^T^ | DSM 117359 | DSM 117358 | DSM 103132 |
| Size (bp) | 2,629,105 | 2,800,819 | 2,536,557 | 2,751,603 | 2,773,270 | 2,671,946 | 2,414,751 | 2,572,492 | - |
| GC-content (%) | 56 | 56 | 56 | 56 | 55 | 53 | 54 | 54 | - |
| Completeness (%) | 99.74 | 99.99 | 99.75 | 99.94 | 99.94 | 99.76 | 99.92 | 99.82 | - |
| Contamination (%) | 0 | 0 | 0 | 0 | 0.1 | 0.21 | 0.01 | 0.19 | - |
| Genes | 2,661 | 2,833 | 2,558 | 2,789 | 2,827 | 2,763 | 2,504 | 2,621 | - |
| CDS | 2,601 | 2,773 | 2,498 | 2,726 | 2,767 | 2,704 | 2,444 | 2,562 | - |
| Functional proteins | 1,590 | 1,602 | 1,540 | 1,582 | 1,659 | 1,612 | 1,515 | 1,549 | - |
| Hypothetical proteins | 1,011 | 1,171 | 958 | 1,144 | 1,108 | 1,092 | 929 | 1,013 | - |
| rRNA (5S; 16S; 23S) | 2; 2; 2 | 2; 2; 2 | 2; 2; 2 | 2; 2; 2 | 2; 2; 2 | 2; 2; 2 | 2; 2; 2 | 2; 2; 2 | - |
| tRNAs | 53 | 53 | 53 | 56 | 53 | 52 | 53 | 52 | - |
| tmRNAs | 1 | 1 | 1 | 1 | 1 | 1 | 1 | 1 | - |
| CRISPR repeats | 3 | 3 | 1 | 3 | 3 | 1 | 1 | 2 | - |
| Assembly accession | CP136425.1 | CP136416.1 | CP136551.1 | CP136417.1 | CP136420.1 | CP136419.1 | CP136421.1 | CP136418.1 | CP017019.1 |
| BioProject | PRJNA996482 | PRJNA996481 | PRJNA996483 | PRJNA996866 | PRJNA996479 | PRJNA998259 | PRJNA996486 | PRJNA996484 | PRJNA329441 |
| BioSample | SAMN36581491 | SAMN36579258 | SAMN36701215 | SAMN36701232 | SAMN36579988 | SAMN36701214 | SAMN36596184 | SAMN36593887 | SAMN05412808- |
| Sequence Read Archive Illumina | SRR25436643 | SRR25436792 | SRR25436649 | SRR25436961 | SRR25444698 | SRR25444695 | SRR25444645 | SRR25444700 | - |
| Sequence Read Archive Nanopore | SRR25436302 | SRR25436905 | SRR25436653 | SRR25436960 | SRR25444699 | SRR25444694 | SRR25445196 | SRR25444701 | - |
| GenBank 16S rRNA | - | - | - | - | OR576718 | - | - | - | - |
| BioProject Phage | - | - | - | - | PRJNA1020657 | - | - | - | PRJNA1020667 |
| BioSample Phage | - | - | - | - | SAMN36579988 | - | - | - | SAMN37527558 |
| Sequence Read Archive Phage | - | - | - | - | SRR26159488 | - | - | - | SRR26160084 |
| GenBank Phage Genome | - | - | - | - | OR602863 | - | - | - | OR602862 |
