# Supplementary material for: Isolation and characterization of novel acetogenic Moorella strains for employment as potential thermophilic biocatalysts
Source: FEMS Microbiol Ecol. 2024 Aug 8;100(9):fiae109. doi: 10.1093/femsec/fiae109 (PMC11328732; doi:10.1093/femsec/fiae109)
Supplement: fiae109_Supplemental_Files [file fiae109_supplemental_files.zip › Supplementary data Table S3.docx]

Table S3: Substrate utilization of the *Moorella* isolates KAM, BGP, COM, MBA and ACPs reporting the highest measured optical density at 600 nm (OD_600_) over the course of 14 days

|  | *M. thermoacetica* KAM | *M. thermoacetica* BGP | *M. thermoacetica* COM | *M. thermoacetica* MBA | *M. carbonis* ACPs^T^ |
| --- | --- | --- | --- | --- | --- |
| L(+)-Arabinose | - | - | - | - | - |
| D(-)-Ribose | - | - | - | - | 0.1^°^ |
| D(+)-Xylose | - | 0.68^**^ | - | - | 1.69^°^ |
| D(-)-Fructose | 1.7^**^ | 1.76^**^ | 1.63^**^ | 2.41^**^ | 1.52^##^ |
| D(+)-Galactose | 0.1^°°^ | 0.1^°°^ | - | 0.13^°°^ | 0.55^°°^ |
| D(+)-Glucose | 0.19^°°^ | 0.88^°°^ | 1.67^°°^ | 0.73^°°^ | 0.97^°°^ |
| D(+)-Mannose | 0.3^°°^ | 0.07^°°^ | - | 0.08^°°^- | 0.74^°^ |
| L(+)-Rhamnose | - | - | - | - | 0.24^°°^ |
| D(-)-Glucuronic acid | 0.7^°°^ | - | - | - | - |
| Glycerol | 0.99^°°^ | - | - | - | - |
| D(-)-Sorbitol | - | - | 1.42^°^ | - | - |
| D(-)-Mannitol | - | - | 0.52^°°^ | - | - |
| *myo*-Inositol | 0.82^##^ | 0.54^**^ | 1.03^**^ | 1.74^**^ | 0.42^°°^ |
| D(+)-Sucrose | 0.24^*^ | 0.17^##^ | 0.18^*^ | 0.07^°°^ | 0.2^*^ |
| D(+)-Cellobiose | - | - | - | - | - |
| D(+)-Trehalose | - | - | - | - | - |
| D(+)-Lactose | - | - | - | - | - |
| D(+)-Maltose | - | - | - | - | - |
| D(+)-Melibiose | - | - | - | - | - |
| D(+)-Raffinose | 0.38^*^ | 0.18^*^ | 0.27^*^ | 0.07^##^ | 0.24^*^ |
| D(+)-Melezitose | - | 0.29^°^ | 0.28^°°^ | weak | 0.09^°°^ |
| Dextran | - | - | - | - | - |
| Dextrin | - | - | - | - | - |
| Starch | - | - | - | - | - |
| Formate | 0.06^°°^ | weak | 0.07^*^ | weak | weak |
| Pyruvate | 0.24^°°^ | 0.22^*^ | 0.45^#^ | - | 0.19^**^ |
| DL-Lactate | 0.25^°°^ | 0.15^°°^ | 0.77^#^ | 0.42^#^ | 0.08^°°^ |
| DL-Malate | - | - | - | - | - |
| Citrate | - | - | - | - | - |
| DMG | weak | weak | weak | weak | weak |
| Betaine | weak | weak | weak | weak | weak |
| Vanillate | 0.06^°°^ | 0.07^##^ | 0.08^**^ | 0.1^°^ | 0.09^##^ |
| Syringate | 0.12^°°^ | 0.15^#^ | 0.1^°^ | 0.12^°°^ | 0.13^#^ |
| Methanol | 0.08^°°^ | 0.22^°°^ | 1^°°^ | 1.14^°°^ | 0.22^°°^ |
| Ethanol | - | - | - | - | - |
| *n*-Propanol | - | - | - | - | - |
| 1,2-Propanediol | - | - | - | - | 0.09^**^- |
| *n*-Butanol | - | 0.07^##^ | - | - | - |
| * = 24 h | ** = 48 h | # = 72 h | ## = 96 h | ° = 120 h | °° = 14 d |
| weak = < OD 0.05 |  |  |  |  |  |
