# Supplementary material for: Isolation and characterization of novel acetogenic Moorella strains for employment as potential thermophilic biocatalysts
Source: FEMS Microbiol Ecol. 2024 Aug 8;100(9):fiae109. doi: 10.1093/femsec/fiae109 (PMC11328732; doi:10.1093/femsec/fiae109)
Supplement: fiae109_Supplemental_Files [file fiae109_supplemental_files.zip › Supplementary data Table S4.docx]

Table S4: Cellular fatty acid analysis of *M. carbonis* and most closely related *Moorella* species. Fatty acid proportions are given in percentage and predominant fatty acids are highlighted in bold.

| Fatty acid | *M. carbonis* (ACPs^T^)  DSM 116161^T^ | *M. stamsii*  DSM 26217^T*^ | | *M. glycerini*  DSM 11254^T*^ | | *M. humiferrea*  DSM 23265^T*^ | | *M. caeni*  DSM 21394^T**^ | |  |
| --- | --- | --- | --- | --- | --- | --- | --- | --- | --- | --- |
| Iso-C_13:0_ | 0.1 | - | | - | | - | | - | |  |
| Iso-C_13:0_ DMA | 0.1 | - | | - | | - | | - | |  |
| C_14:0_ | 5.0 | 0.71 | | 2.07 | | 0.97 | | 2.71 | |  |
| C_14:0_ DMA | 0.2 | - | | - | | - | | - | |  |
| C_14:0_ 8,11-dimethyltetradecanoic acid | 1.1 | - | | - | | - | | - | |  |
| C_15:0_ | - | 1.86 | | - | | - | | - | |  |
| Iso-C_15:0_ | **40.1** | **26.18** | | **37.62** | | **20.58** | | **47.36** | |  |
| Iso-C_15:0_ DMA | **23.1** | **15.11** | | **18.15** | | 1.60 | | **20.27** | |  |
| Iso-C_15:0_ ALDE | 2.2 | - | | - | | - | | - | |  |
| Anteiso-C_15:0_ | - | 2.23 | | - | | - | | - | |  |
| C_16:0_ | 5.4 | 7.11 | | **10.56** | | **21.65** | | 9.1 | |  |
| C_16:0_ DMA | 1.6 | 3.35 | | 2.50 | | 3.29 | | 1.66 | |  |
| C_16:0_ DMA (x-methyl) | 4.1 | - | | - | | - | | - | |  |
| Iso-C_16:0_ | - | 5.39 | | - | | - | | - | |  |
| C_17:0_ DMA | - | - | | 1.15 | | 2.99 | | 5.79 | |  |
| C_17:0_ cyclopropane | - | - | | - | | 2.21 | | 3.04 | |  |
| C_17:0_ DMA (x-methyl) | 1.0 | - | | - | | - | | - | |  |
| Iso-C_17:0_ | 3.7 | 6.52 | | **11.30** | | **21.85** | | 5.45 | |  |
| Iso-C_17:0_ DMA | 8.9 | - | | - | | - | | - | |  |
| Anteiso-C_17:0_ | - | 2.15 | | - | | - | | - | |  |
| Anteiso-C_17:0_ DMA | - | 2.27 | | - | | - | | - | |  |
| C_18:0_ | 0.3 | 1.38 | | 1.79 | | **13.49** | | 1.76 | |  |
| C_18:0_ DMA | 0.1 | - | | - | | 1.12 | | - | |  |
| C_18:0_ DMA (x-methyl) | 3.0 | - | | - | | - | | - | |  |
| C_18:1_ ∆9 | - | - | | - | | 0.90 | | - | |  |
| C_19:0_ cyclo-11,12 DMA | - | - | | 1.44 | | - | | - | |  |
| * data from (Alves *et al.* 2013) | | | | | | | | | | |
| ** data from (Vecchini Santaella, Sousa and Stams 2023) | | | | | | | | | | |
| DMA – dimethyl acetal  ALDE - aldehyde |  | |  | |  | |  | |  | |
| x-methyl – position of methyl group unclear |  | |  | |  | |  | |  | |
