# Supplementary figures and images for: Isolation and characterization of novel acetogenic Moorella strains for employment as potential thermophilic biocatalysts
Source: FEMS Microbiol Ecol. 2024 Aug 8;100(9):fiae109. doi: 10.1093/femsec/fiae109 (PMC11328732; doi:10.1093/femsec/fiae109)

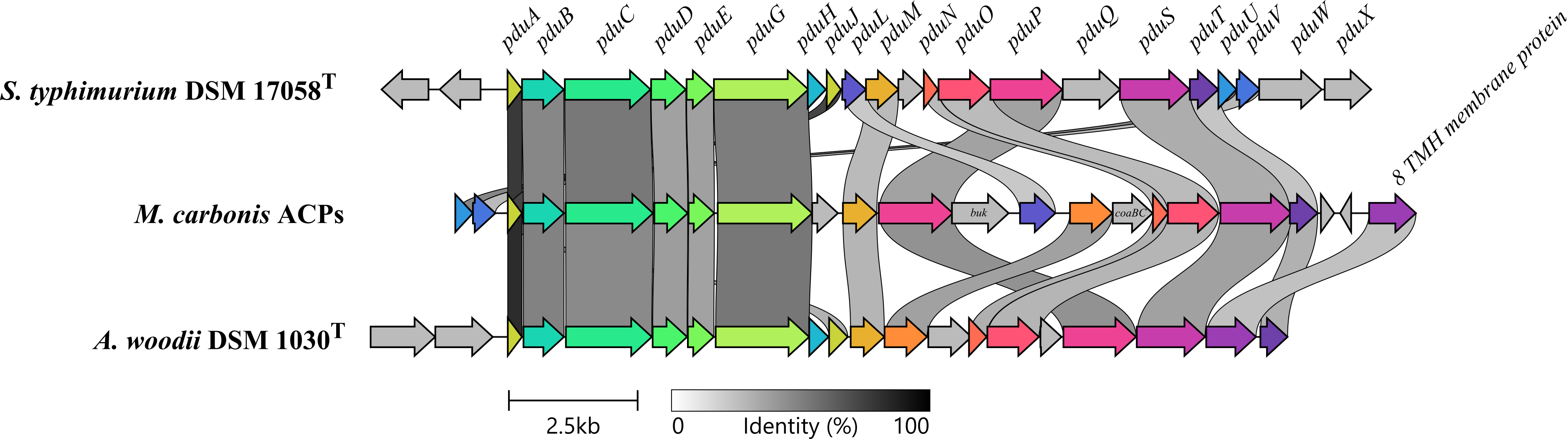

Supplement: fiae109_Supplemental_Files [file fiae109_supplemental_files.zip › supp data FigS1.tif]

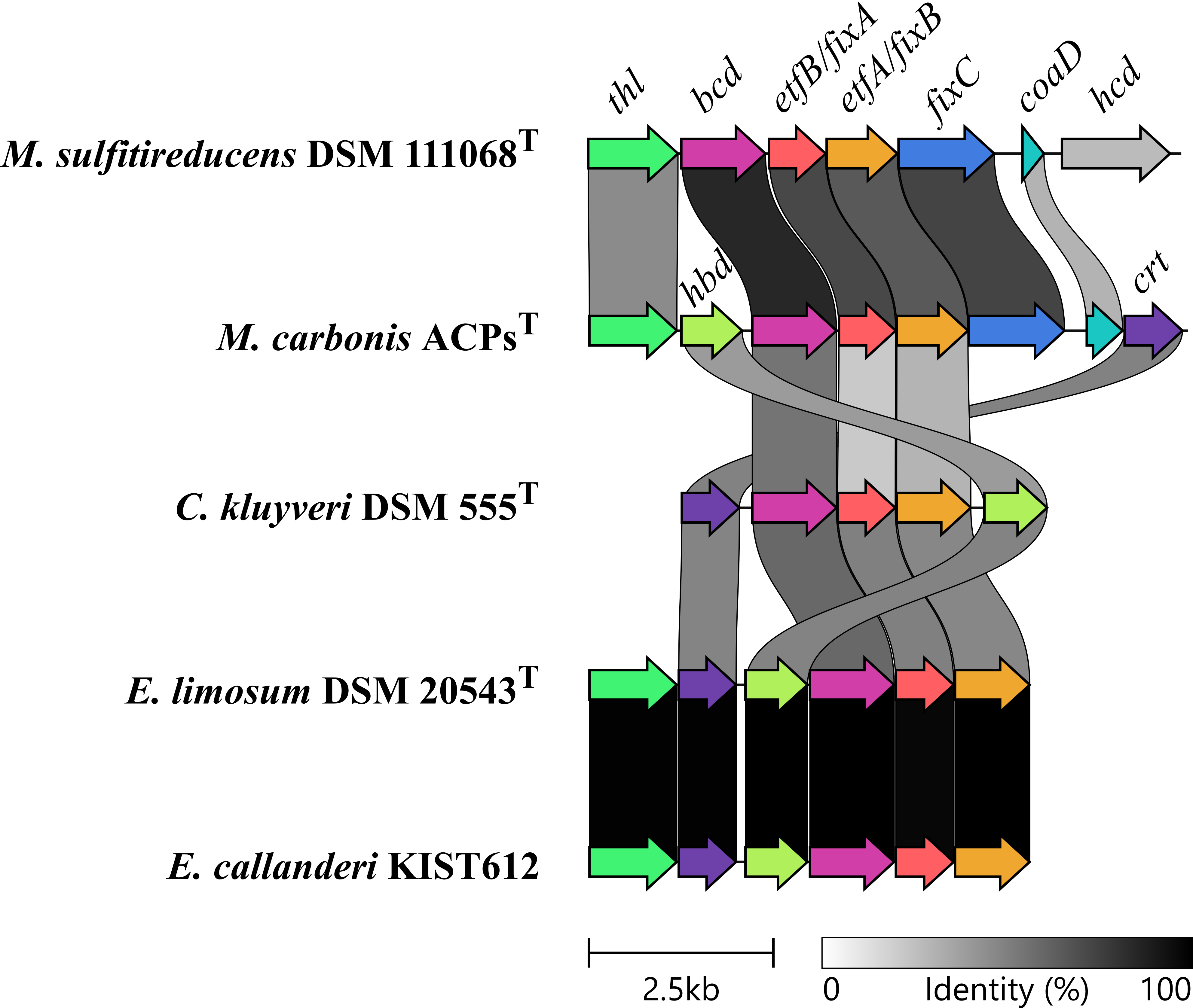

Supplement: fiae109_Supplemental_Files [file fiae109_supplemental_files.zip › supp data FigS2.tif]
